# Small RNA Based Genetic Engineering for Plant Viral Resistance: Application in Crop Protection

**DOI:** 10.3389/fmicb.2017.00043

**Published:** 2017-01-23

**Authors:** Annum Khalid, Qingling Zhang, Muhammad Yasir, Feng Li

**Affiliations:** Key Laboratory of Horticultural Plant Biology, Ministry of Education, College of Horticulture and Forestry Sciences, Huazhong Agricultural UniversityWuhan, China

**Keywords:** siRNA, miRNA, crop protection, viruses, vegetable, fruit, staple food, genetic engineering

## Abstract

Small RNAs regulate a large set of gene expression in all plants and constitute a natural immunity against viruses. Small RNA based genetic engineering (SRGE) technology had been explored for crop protection against viruses for nearly 30 years. Viral resistance has been developed in diverse crops with SRGE technology and a few viral resistant crops have been approved for commercial release. In this review we summarized the efforts generating viral resistance with SRGE in different crops, analyzed the evolution of the technology, its efficacy in different crops for different viruses and its application status in different crops. The challenge and potential solution for application of SRGE in crop protection are also discussed.

## Impact of Viral Disease on Crop Production

Modern plant virology commenced at the end of 19th century with the research on tobacco mosaic disease done by Russian scientist Dmittrii Iwanowski and Dutch microbiologist Martinus Beijerinck who discovered the causal agent was much smaller in size compared to other microbes because it can pass bacteria-proof filter candle (Roger, 2014). Later on, this causal agent was termed tobacco mosaic virus (TMV) and became the first virus to be defined. Since then, numerous viruses infecting bacteria, fungi, plants and animals were discovered. Currently more than 6,000 viruses were identified according to the Ninth Report of International Committee on Taxonomy of Viruses, of which about 1,300 are plant viruses (King et al., 2012; Roger, 2014).

Plant viruses impose serious threats to wide range of crops in modern agriculture and it is estimated that economic loss caused by viral pathogen ranks the second compared to those caused by other pathogens (Simon-Mateo and Garcia, 2011). Depending on its nature, some viruses can have very broad host range. For example, tomato spotted wilt virus (TSWV) is reported to infect more than 1000 plant species in 85 families, including many vegetables, peanut, and tobacco (Sherwood et al., 2003) and cucumber mosaic virus (CMV) can infect more than 1200 plant species in 100 families, including many vegetables and ornamentals (Zitter and Murphy, 2009).

Plant viral disease significantly reduces crop quality and yield. It has been estimated that potato leaf role virus (PLRV) resulted in 20 million ton losses in potato production worldwide annually (Kojima and Lapierre, 1988). In most subtropical and tropical areas, tomato leaf curl virus (ToLCV) can cause complete economic loss in a tomato field (Czosnek and Laterrot, 1997). Since late 1980s in central and east Africa, cassava crops in almost 12 different countries were damaged due to cassava mosaic disease caused by cassava mosaic virus (CsMV) (Legg et al., 2011). In Southeast Asia, Rice tungro virus has been estimated to cause an annual loss of 5–10% of the rice yield (Dai and Beachy, 2009). Broad range of plants including tobacco, tomato, and peanuts has been infected by TSWV (Sherwood et al., 2003) and as a result, the annual economic losses due to this virus are projected to be one billion dollars worldwide (Roger, 2014).

## Virus Infection and RNA Silencing in Plants

Due to their devastating threat to crop production, plant viruses has been studied extensively since the first virus, TMV, was discovered. The outcome of a virus infection on a plant is determined both by the genotype of the virus and that of the plant. The plant genetic architecture conferring resistance/tolerance to viruses usually includes so called recessive resistance and active defense. Recessive resistance is usually conferred by lacking positive host factors for virus propagation and accounted by many excellent reviews (Diaz-Pendon et al., 2004; Truniger and Aranda, 2009; Wang and Krishnaswamy, 2012; Nicaise, 2014). In contrast to the passive defense model, plants can also actively attack viruses upon recognition of infection with a plethora of chemical and enzymatic arsenals (Vlot et al., 2009; Ding, 2010; Fu and Dong, 2013; Alazem and Lin, 2015). Among the many active defense mechanisms, RNA silencing was discovered more recently but attracted the most attention in the past decade in plant–virus interaction studies (Li and Ding, 2006; Mlotshwa et al., 2008; Ding and Lu, 2011; Baulcombe, 2015; Carbonell and Carrington, 2015).

**FIGURE 1:**
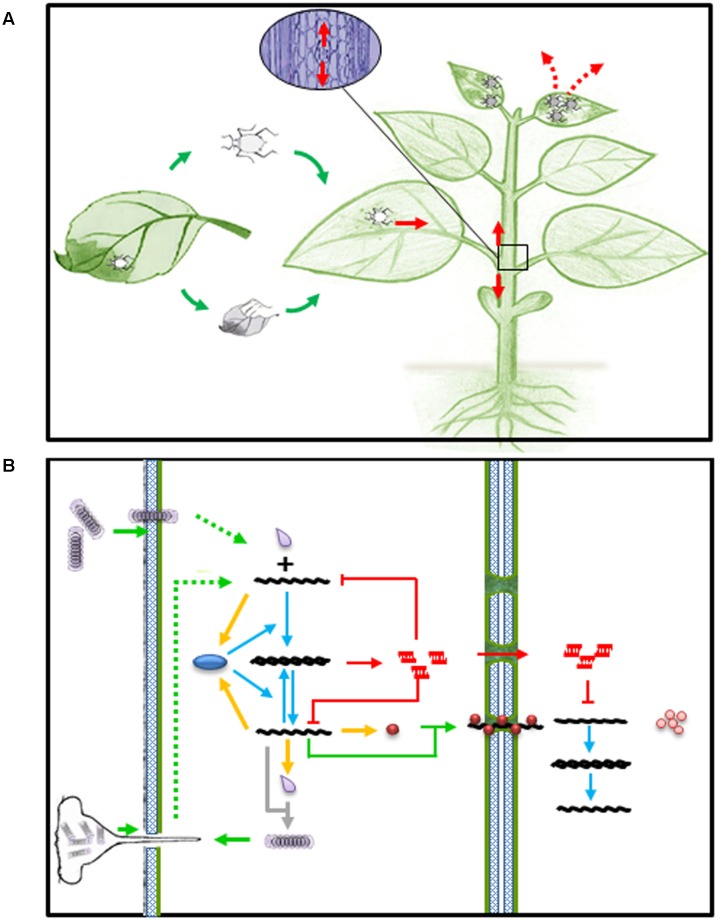
**Viral infection and RNA silencing in plants.** **(A)** Virus entry (green arrows), spread (read arrows) and exit (read dashed arrows) in host plant. **(B)** Virus entry and spread (green arrows) in plant cell. Green dashed arrows represent disassembly of virion upon entry into plant cell. Yellow arrows represent expression of viral products, such as replicase (blue oval), movement protein (brown ball), and capsid protein (gray droplet). Blue arrows represent transcription of viral RNAs. Gray arrow depicts virion assembly from newly synthesized capsid and genomic RNA. Red arrows and lines represent activation of small RNA mediated intra and inter-cellular immunity.

Viruses are obligate intracellular parasites and complete their life cycle in living host cells. Plant viruses usually enter plant cells through wounds made by insect vectors or mechanic rubbing, replicate in the initial infected cells, move from cell to cell via plasmadosmata, and spread via phloem into newly emerged young tissue and organ, where they cause disease phenotype and became ready to exit and infect other host plants (**Figures 1A,B**). At the molecular and cellular level, once a virus particle, such as TMV, gets into a host cell, it has to be disassembled to release its genomic (g)RNA. The gRNA then serves as mRNA to produce viral replicase protein, which in turn transcribe gRNA into a complementary (c)RNA and further transcribe more gRNA and subgenomic (sg)RNA using cRNA as template. The amplified gRNA can participate in at least four possible pathways: replication, translation, cell-to-cell movement and assembly (**Figure 1B**).

In the middle 1980s, Sanford and Johnston formulated an elegant concept of pathogen derived resistance (PDR) that “Key gene products from the parasite, if present in a dysfunctional form, in excess, or at the wrong developmental stage, should disrupt the function of the parasite while having minimal effect on the host” (Sanford and Johnston, 1985). It is assumed that all viral activities during infection require that viral proteins interact with different host factors in a proper temporal and spatial manner. Thus PDR was applied to engineer viral resistance in plants by transforming plants with various viral genes since late 1980s and led to successful development of viral resistant crops for commercial application (Baulcombe, 1996). The first PDR in plants was demonstrated by transformation of tobacco plants with TMV coat protein gene (Abel et al., 1986). Numerous attempts were then conducted to generate viral resistance in plants through expression of viral proteins from transgene and in several cases it is consistent with the original idea of PDR, while in many other cases they were not explained by protein based PDR rather led to the discovery of small RNA based RNA silencing mechanism (Baulcombe, 1996; Palukaitis, 2011).

RNA silencing refers to small interfering (si)RNAs or micro(mi)RNAs mediated sequence specific gene silencing mechanisms, which play important role in antiviral defense, development, and maintenance of genome integrity (Ding, 2000; Vance and Vaucheret, 2001; Baulcombe, 2004; Chen, 2012). In plants, several key protein families are involved in RNA silencing, including Dicer-like (DCL), Argonautes (AGO), and RNA-dependent RNA Polymerase (RDR). DCL proteins are type III RNases that process dsRNA or hairpin RNA into siRNA or miRNA, respectively, of 20- to 24-nt long with 2-nt 3′ overhang. AGO proteins are endonucleases that form RNA-induced silencing complex (RISC) with siRNAs or miRNAs. RISC can bind to target mRNA or non-coding RNA by sequence complementarity via its containing siRNA/miRNA, and then silence the target gene expression by cleaving target mRNA and rendering its degradation, or recruiting cofactors and inhibiting mRNA translation, or recruiting DNA and histone modifiers and inhibiting the transcription of target gene. RDR proteins transcribe single-stranded RNA into dsRNAs which is further processed into siRNA by DCL protein. While DCL and AGO proteins present in all organisms where RNA silencing operates, RDR only presents in fungi, plants and very few animals, such as worms and amphioxus (Wassenegger and Krczal, 2006).

In plants DCL, AGO, and RDR are gene families containing multiple members and each functions in different parallel pathways. In *Arabidopsis* many studies have shown that DCL2, DCL4, AGO1, AGO2, RDR1, and RDR6 are the major components in antiviral RNA silencing (Carbonell and Carrington, 2015; Zhang et al., 2015). It is suggested that the double stranded replicative intermediates of RNA viruses or structured single stranded viral RNA can be processed by plant DCL4 or DCL2 into primary viral siRNAs (Voinnet, 2005; Bouche et al., 2006; Deleris et al., 2006; Ding and Voinnet, 2007). These primary viral siRNAs form RISC with AGO1 or AGO2, which target viral mRNAs for degradation. The RDR1 and RDR6 may use the cleaved viral RNA as substrate to synthesize dsRNA, which is further processed by DCL2 and DCL4 into secondary viral siRNA. These secondary viral siRNAs enhance antiviral RNA silencing by forming RISC complexes and targeting viral mRNA in the initial infected cells, or alerting the neighboring cell as well as the systemic tissue by the cell-to-cell and systemic movement via plasmadesmata and phloem respectively.

Since generation of dsRNA is a general feature during the replication and gene expression of various types of virus, dsRNA triggered RNA silencing is considered a pathogen molecular pattern (PAMP)- triggered immunity (PTI) in plants (Ding, 2010). In line with the zigzag model of the pathogen–host co-evolution (Jones and Dangl, 2006), virus that can overcome RNA silencing based PTI, usually encode effector that suppresses RNA silencing, which is termed viral suppressor of RNA silencing or VSR (Li and Ding, 2006). Many viruses encode different VSR proteins that suppress RNA silencing using diverse mechanisms (Li and Ding, 2006; Burgyan and Havelda, 2011; Jiang et al., 2012; Csorba et al., 2015).

## RNA Silencing Mechanisms and their Viral Targets in Crop Improvement

**FIGURE 2:**
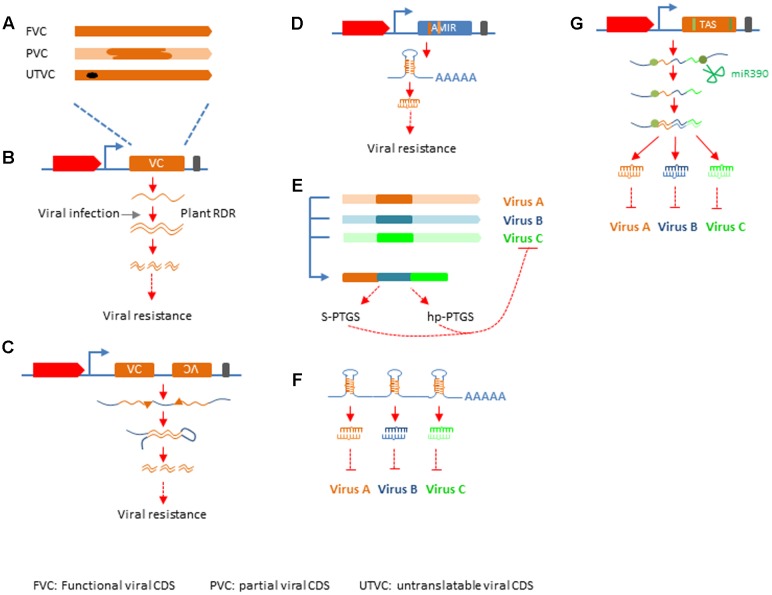
**Silencing mechanisms applied in crop protection.** **(A)** Different types of viral sequences used in genetic engineering. FVC, functional viral CDS; PVC, partial viral CDS; UTVC, untranslatable viral CDS. **(B)** S-PTGS, top: structure of silencing construct with red block representing plant promoter, yellow block representing inserted viral sequences, black bar representing transcription terminator. **(C)** hp-PTGS, top: structure of silencing construct as depicted in **(B)**, except there are two viral sequences one in sense and the other in antisense orientation. **(D)** AMIR-PTGS, the structure of AMIR construct is similar to that in **(B,C)**, except that the blue block represent a backbone sequences of a natural miRNA and the dark yellow bar within the blue block depict mature miRNA sequence designed to target viral genome and the light yellow bar represents miRNA star. **(E)** Strategy to generate multiple-viruses resistance in S-PTGS and hp-PTGS. The yellow, blue, and green bars represent different viral sequences. The forth bar with different colors represents the chimeric viral sequences used in S-PTGS and hp-PTGS. **(F)** Cluster of AMIRs for multiple-viruses resistance. **(G)** TAS for multiple-viruses resistance. The TAS gene structure is similar to that described in **(A)**, except the blue block represents natural TAS3 backbone. The green bar in the gene structure and green dots in transcript lines represent miR390 binding sites.

RNA silencing has been deployed in crop improvement for viral resistance along the way it has been discovered. Successful resistance was achieved with either full length cDNA encoding functional viral products, or partial, or mutated viral cDNA (**Figures 2A**; **Supplementary Table S1**). These efforts can be categorized into four groups based on the mechanisms by which antiviral silencing is activated, sense gene induced post-transcriptional gene silencing (S-PTGS), hairpin RNA induced PTGS (hp-PTGS), artificial miRNA induced PTGS (AMIR), and trans-acting siRNA induced PTGS (TAS) (**Supplementary Table S1**; **Figures 2B–E**).

**S-PTGS** was practiced very early and very successful in the effort generating viral resistance (Gielen et al., 1991; Fitch et al., 1992; Lindbo et al., 1993). Inspired by the “PDR” hypothesis, researchers tried to generate viral resistance by overexpression of a viral protein in these efforts. However, the mechanism was turn out to be RNA mediated post-transcriptional gene silencing in many cases (Lindbo et al., 1993). The silencing state can be achieved before or after viral infection. In either case, it requires plant RDR protein to transcribe the overexpressed viral sequences into dsRNA, which is processed into siRNA to enhance the antiviral silencing mediated by siRNA derived from viral replication (**Figure 2B**) (Mourrain et al., 2000; Ding and Voinnet, 2007). Protection by S-PTGS type transgene can vary significantly among different lines transformed with the same construct (**Supplementary Table S1**). Transgenic lines that accumulated high level of viral siRNA and established silencing state in absence of viral infection usually are immune or highly resistant to viral infection (Guo et al., 1998; Masmoudi et al., 2002). On the contrary, transgenic lines express viral transcripts before viral invasion showed variable degree of resistance, ranging from susceptible, delaying in symptom expression, recovery to resistant (Lindbo et al., 1993; Sivamani et al., 2002; Zanek et al., 2008; Reyes et al., 2011). Since early 1990s, expression of antisense RNA was also tested in genetic engineering for viral resistance and various degree of resistance was obtained (Prins et al., 1996, 1997). Silencing mechanism behind these approaches is similar to that of S-PTGS and thus it is categorized as AS-PTGS. Both S-PTGS and AS-PTGS are considered first generation of small RNA based genetic engineering (SRGE) technology for viral resistance which was invented before the RNA silencing mechanism was well-understood and are still widely used till recently (**Supplementary Table S1**).

**Hp-PTGS** is the second generation technology developed after dsRNA was recognized as the trigger of RNA silencing. In these practices, researchers constructed silencing vectors with pieces of both sense and antisense viral cDNA under control of plant promoters and terminators. When transformed into plants, these constructs produce transcripts that can fold into dsRNA due to the complementarity of sense and antisense viral sequences in it. The dsRNA is then processed into siRNAs and confers resistance/immunity to cognate viruses (**Figure 2C**). The first example of hp-PTGS mediated viral immunity was done in tobacco against PVY, which was published at the same year as the seminar paper showing dsRNA is the trigger of RNAi in worms (Fire et al., 1998; Waterhouse et al., 1998). Later on this technology was applied in many crops against diverse viruses and in most cases the degree of resistance to target virus in the transgenic plants was high to immune (Kalantidis et al., 2002) (**Supplementary Table S1**).

**AMIR** is considered the third generation technology developed very recently. In the first two generations of small RNA technology, the mature small RNAs that function in viral immunity are not predefined. Since loading of small RNA into the silencing effector AGO proteins requires certain sequence features in those small RNAs (Czech and Hannon, 2011), many small RNAs generated by the first two generation technology may not feed into the effectors. Natural miRNAs are released from well-defined secondary structure in their pri-miRNA transcripts. In the AMIR approach, the mature miRNA sequences in a natural miRNA primary transcript were replaced with specific RNA sequences that are complementary to target viruses and have favorable features for RISC loading, thus to create an artificial miRNA gene. When transformed into plants, the AMIR gene was transcribed and processed into mature miRNA with the designed sequences by the cellular miRNA biogenesis machinery to confer specific virus resistance (**Figure 2D**). The proof-of-concept studies for AMIR mediated viral resistance were reported in *Arabidopsis* and tobacco nearly 10 years ago (Niu et al., 2006; Qu et al., 2007), while its application in crop improvement is very limited and currently only two cases in tomato were reported besides those aforementioned (Zhang et al., 2011a; Vu et al., 2013) (**Supplementary Table S1**).

**Mechanisms for multiple virus resistance** were developed since the first generation technology. And there were at least five strategies developed to achieve this goal. The first one was to generate silencing constructs with multiple transcription units each targeting a distinct virus using S-PTGS mechanism (Prins et al., 1995; Shin et al., 2002). The second way was developed by Arif et al. (2009), in which double resistance was obtained by co-transformation with two constructs each target different virus by S-PTGS. In the third way, silencing construct was made with multiple inverted-repeat sequences derived from conserved viral sequences. Each IR structure can produce a dsRNA that can induce hp-PTGS against cognate virus (Zhang et al., 2011b). The forth, also a more widely applied strategy is to piece together partial gene fragments from different viruses first and then generate S-PTGS or hp-PTGS construct with chimeric viral sequences, which will produces siRNAs that target all intended viruses (**Supplementary Table S1**; **Figure 2E**) (Bucher et al., 2006; Liu et al., 2007; Kung et al., 2009; Lin et al., 2012). Transgenic plants were obtained by this strategy with complete resistance up to six different viruses (Liu et al., 2007). The fifth one is to generate cluster of artificial miRNA precursors and express it from one construct to generate multiple functional miRNAs targeting different viruses (**Figure 2F**). The sixth strategy was using artificial trans-acting siRNA gene to express multiple tasiRNAs targeting different viruses. A stretch of synthetic sequences consists of multiple 21-nt short sequences that complementary to target viruses is inserted between a 3′-cleavable and a 5′-non-cleavable miR390 binding sites to create an artificial TAS transcript. When it is expressed in transgenic plants, the artificial TAS transcript was cleaved by miR390 and the 5′ cleavage product containing the viral sequences is turned into dsRNA by plant RDR6 and diced from its 3′ end successively by DCL4 to release 21-nt tasiRNA that will target cognate viruses (**Figure 2G**). The efficacy of TAS-mediated multiple-viruses resistance has been demonstrated in *Arabidopsis* (Chen et al., 2016).

**FIGURE 3:**
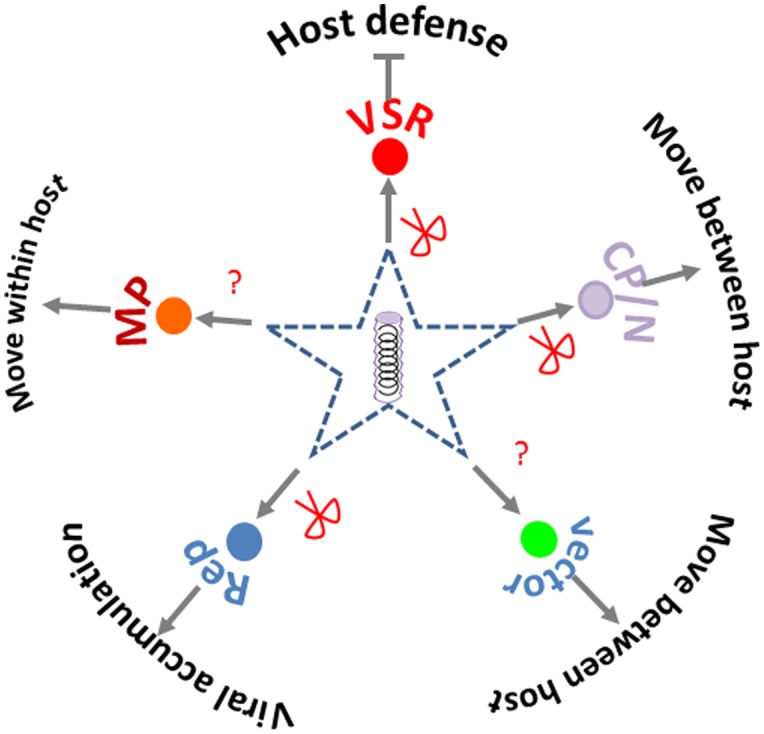
**Silencing targets chosen in crop protection.** The red scissors point to the viral products (functions) that had been targeted by small RNA based genetic engineering. The question marks point to the viral or vector function yet to be reported as targets for crop protection.

**Viral targets** for SRGE include both the virus and the genes or region within a virus that are targeted by small RNAs produced by the transgene. In terms of number of studies, Potyviruses, Tospoviruses, Closteroviruses, and Geminiviruses are among the most studied virus genera (**Supplementary Table S1**). Viral coat protein or nuclear capsid protein are the most frequently chosen targets for SRGE; viral replicase or replication associated proteins, and VSR protein are also frequently used (**Supplementary Table S1**). All these targets provide essential function for virus life cycle (**Figure 3**). Besides targeting the coding region, the untranslated region (UTR) in viral genome is also targeted for efficient antiviral silencing (Duan et al., 2008), due to its key role in viral replication and viral mRNA translation.

**Choice of promoter** in making silencing constructs. In many cases 35S promoter from *Cauliflower mosaic virus* was used to drive the expression of silencing transcripts to produce siRNA or miRNA targeting viruses. The 35S promoter is active in most vegetative tissue and drives gene expression constitutively. Phloem tissue is the highway for viral systemic spread within the plants. In a study both phloem-specific promoter and 35S promoter were tested to drive the expression of silencing genes and the 35S promoter driven construct provided better resistance (Ehrenfeld et al., 2004). Expression of virus-targeting small RNA constitutively in all cell types may provide second line of defense in case virus breaks the defense in phloem and evade into newer tissue.

## Application Status of SRGE in Crop Protection

**FIGURE 4:**
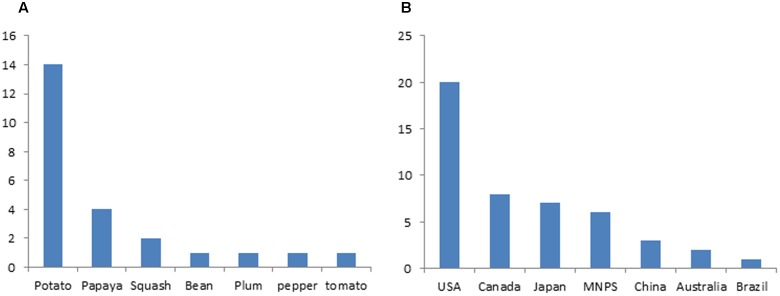
**Application status of small RNA based genetic engineering in crop protection.** **(A)** Number of small RNA based transgenic crop varieties that are approved for commercial release. **(B)** Number of small RNA based transgenic crop varieties in different countries that are approved for commercial release.

Small RNA based genetic engineering has been applied in engineering viral resistance for many crops, including major crops of staple food, vegetables, fruits ornamentals, and some cash crop (**Supplementary Table S1**). *Nicotiana benthamiana* has been widely used as a model species to study the efficacy of constructs for silencing the intended virus (**Supplementary Table S1**). Stable transgenic plants for a variety of crops were generated expressing small RNAs in different ways and their reactions to targeted viruses were tested in both laboratory and field condition (**Supplementary Table S1**). In some studies, the durability of resistance was tested for many generations (Wang et al., 2001, 2016; Liu et al., 2007; Cruz et al., 2014; Faria et al., 2014). According to the International Service for the Acquisition of Agri-Biotech Applications (ISAAA) website, dozens of transgenic crops resistance to virus generated with SRGE were approved for commercial release (**Supplementary Table S2**). Potato and the United States ranks the top among different crops and countries, respectively, in terms of number of lines approved (**Figure 4**). All these commercially released crops were developed based on the first generation SRGE technology.

**Papaya** provided the first successful example for tackling down the virus threats in agriculture with the SRGE. Papaya is an important tropical fruit with high nutritional value and economic significance. But the papaya industry was nearly destroyed in some regions by Papaya ringspot virus, a potyvirus with positive sense single strand RNA genome, in early 1990s (**Supplementary Table S1**) (Ferreira et al., 2002). Lack of natural resistance resources and effective disease management strategy made it necessary to the development of transgenic PRSV-resistant papaya and the effort was started late 1980s by Maureen Fitch, Dennis Gonsalves and colleague with the “PDR” approach (Gonsalves, 2006). PRSV-resistant papaya was soon obtained by expressing viral CP through transgene (Fitch et al., 1992) and commercially released in 1998 in Hawaii (Gonsalves, 2006). Due to the specificity of small RNA silencing mediated immunity, the transgene developed in Hawaii did not confer resistance to PRSV strain in Asia and new transgenic papaya lines were developed later with CP genes from local viral strain (Bau et al., 2003). Overcoming of resistance by more virulent PRSV strain was observed and new resistant transgenic papaya was obtained by targeting the viral HcPro protein that suppresses small RNA mediated immunity (Kung et al., 2015). Currently, there are four commercial transgenic papaya lines approved with three in USA and one in China (**Supplementary Table S2**; **Figure 4A**).

**Banana, Citrus and Plum**, banana is the largest tropical fruit and BBTV is the most serious viral pathogen for banana cultivation worldwide. BBTV-resistant transgenic banana was developed with hp-PTGS mechanism targeting Rep gene (Shekhawat et al., 2012; Elayabalan et al., 2013). Citrus is a high value fruit crop in international trade for both fresh fruits and juice market. CTV is the most economically important and damaging virus of citrus tree. CTV-resistant citrus was obtained with hp-PTGS targeting multiple VSR genes in the virus genome (Soler et al., 2012) while targeting single VSR is not effective (Batuman et al., 2006). Early efforts with S-PTGS mechanisms also did not work very well in citrus (**Supplementary Table S1**). Neither banana nor citrus transgenic lines resistant to viruses were approved for commercial release. Plum is one of the oldest domesticated fruit with versatile uses. Plum pox virus is the major viral pathogen of plum. S-PTGS mediated resistance against Plum pox virus was first demonstrated in *N. benthamiana* (Guo et al., 1998; Wittner et al., 1998) and later in Plum (Scorza et al., 2001). PPV-resistant plum was also obtained with hp-PTGS mechanism targeting CP gene (Hily et al., 2007; Ravelonandro et al., 2014). The S-PTGS based PPV-resistant plum was approved for commercial release in US (**Supplementary Table S2**).

**Squash, cucumber, and watermelon** are common vegetables and fruits belonging to the *Cucubitaceae* family, which suffer from a variety of viral pathogen (Romay et al., 2014). SqMV-resistant squash and CFMMV-immune cucumber were generated by S-PTGS targeting viral CP and Rep gene respectively (Pang et al., 2000; Gal-On et al., 2005). Multiple-viruses resistant Oriental melon and Watermelon were recently reported using S-PTGS with chimeric viral CP sequences (Wu et al., 2010; Lin et al., 2012). PRSV-resistant Cantaloupe was obtained by hp-PTGS mechanism (Krubphachaya et al., 2007). None of these transgenic cucurbita crops were approved for commercial release. Instead, two Squash transgenic lines resistant to CMV and ZYMV were approved for release in Canada and US (**Supplementary Table S2**).

**Potato, tomato, and pepper** are important vegetables belonging to *Solanaceae* family and potato is also a very important staple food crop. These crops suffer from a variety of plant viruses and a number of efforts to generate viral resistance with SRGE were reported (**Supplementary Table S1**). Doreste et al. (2002), Nunome et al. (2002), and Shin et al. (2002) PVX-resistant potato, CMV-resistant tomato and pepper with dual resistance to ToMV and CMV were obtained by means of S-PTGS. Since then PLRV-, PVX-, and PVY-immune potato was developed with hp-PTGS mechanism targeting PLRV-CP, PVX-CP, and PVY-HcPro simultaneously (Arif et al., 2012). TYLCV-immune tomato was also generated with both S-PTGS and hp-PTGS mechanism targeting viral Rep gene (Antignus et al., 2004; Fuentes et al., 2006). Currently, there are 14 transgenic potato lines approved for commercial release in US and other countries and all are developed by the Monsanto Company. One pepper and one tomato line were developed by Peking University and approved for commercial release in China (**Supplementary Table S2**).

**Maize, Wheat, Rice, and Cassava** are the major staple food crop and supported calorie consumption for most of the human population. Maize streak virus (MSV) and maize dwarf mosaic virus (MDMV) impose the most frequent viral threat to Maize production. Transgenic maize resistant to MDMV was generated with hp-PTGS mechanism targeting P1 and CP (Zhang et al., 2010, 2013; Zhang Z.Y. et al., 2011) whereas MSV-resistant transgenic maize was created with S-PTGS mechanism targeting viral Rep gene (Shepherd et al., 2007). Transgenic wheat resistant to Wheat streak mosaic virus was created with all three generations of SRGE and newer ones appeared to provide better protection (Sivamani et al., 2000; Fahim et al., 2010, 2012). The most important viral threat for rice production came from *Phytoreoviruses*, *Tenuiviruses*, *Tungroviruses*, and *Waikavirus*, such as RBSDV, RSV, RTBV, and RTSV (**Supplementary Table S1**). These viral pathogens caused significant losses in rice production in Asia and many resistant transgenic rice lines were generated using hp-PTGS mechanism (Ma et al., 2004; Tyagi et al., 2008; Roy et al., 2012; Sasaya et al., 2014). Some of the resistance traits had been introgressed into cultivated rice varieties (Roy et al., 2012; Valarmathi et al., 2016). Cassava is an important food crop in Africa and *Begmoviruses*, such as ACMV and SLCMV, caused severe problem in Cassava cultivation (Taylor et al., 2004). Initially, ACMV-resistant cassava was created with S-PTGS targeting AC1 gene (Chellappan et al., 2004). Since, ACMV is a DNA virus and its gene expression takes place on viral mini-chromosome structure, viral resistant transgenic cassava was also obtained using hairpin RNA construct targeting the viral promoter for transcriptional gene silencing (hp-TGS) (Vanderschuren et al., 2007). Though, the effectiveness of the transgenic viral resistance has been tested in field trial for many generations (Shimizu et al., 2011; Cao et al., 2013; Wang et al., 2016), currently no SRGE based staple food crop was reported for commercial release.

**Peanut, Soybean, and common bean** are rich in fatty acid, protein and other nutrients, important for everyday diet, and are all from *Fabaceae* family. PStV- and TSV-resistant peanuts were generated successfully with S-PTGS mechanism targeting (Higgins et al., 2004; Mehta et al., 2013), however, this strategy did not work very well for making transgenic peanut against *Tospoviruses*, such as PBNV and TSWV (**Supplementary Table S1**). Soybean mosaic virus is the most important viral pathogen to soybean cultivation and several transgenic lines resistant to this virus were generated by hp-PTGS and S-PTGS, targeting HcPro and CP, respectively (Wang et al., 2001; Gao et al., 2015). Multiple-viruses resistant soybean was also generated by expressing multiple short hairpin targeting Rep of AMV, BPMV, and SMV (Zhang et al., 2011b). BGMV-partial-resistant common bean was initially generated with S-PTGS mechanism targeting CP and completely resistant transgenic line was recently obtained using hp-PTGS targeting AC1 gene (Faria et al., 2006, 2014; Aragao et al., 2013). BGMV-resistant common bean was approved for commercial release in Brazil (**Supplementary Table S2**) while no commercial release of SRGE based viral resistant peanut and soybean were reported.

Tobacco including *Nicotiana tabacum* and *N. benthamiana* were widely used as model plants to study the efficacy of SRGE against various viruses infecting crops (**Supplementary Table S1**) due to their easiness in transformation. However, result obtained from tobacco is not always consistent with that in the intended crop (Batuman et al., 2006). It is possible that certain virus may be more virulent in its native host due to better fitness. Since small RNA mediated silencing is usually dose dependent, this problem can be solved by targeting multiple viral genes in one construct and screen multiple transgenic lines for better resistance (Soler et al., 2012). It is also important to choose a proper promoter to drive the silencing construct expression in targeted crop as it is shown that small RNA subcellular localization affect antiviral efficiency (Ehrenfeld et al., 2004). Another issue in testing the resistance considered is the method of viral inoculation. Viral saps and *Agrobacterium*-mediated infiltration is widely used for virus inoculation as a routine technique in the lab. It was reported that transgenic tomato showed better resistance when infected via insect than by Agro-infiltration (Antignus et al., 2004), which may due to lower viral dosage in vector mediated infection than in Agro-infiltration. Thus choosing proper viral dosage is important in characterization of transgenic lines.

## Challenges and Future Aspects

Early application of the first generation SRGE involves expression of functional viral products, which raises concerns to the human health and the environment. These concerns were well-addressed in the application of PRSV CP transgenic papaya (Fuchs and Gonsalves, 2007). In the newer generation of SRGE technology, only short stretches of viral sequences were expressed and no viral protein product will be expressed in any part of the transgenic crop, thus completely dismiss the concerns, such as heterologous encapsidation, recombination and synergism. However, there still exist real challenges for application of even the second and third generation SRGE.

Crop plants are often subjected to mixed viral infection. VSR from untargeted virus can suppresses the small RNA mediated silencing thus breaks the immunity to SRGE targeted virus (Savenkov and Valkonen, 2001; Simon-Mateo et al., 2003). For the targeted viruses, some isolate has stronger VSR that can break immunity conferred by SRGE (Kung et al., 2015). To solve these problems, multiple virus resistance can be explored with the second and third SRGE technology. It is also necessary to target multiple-genes within one virus to achieve stronger resistance.

Oomycete pathogen was shown to deliver effector into plant cells to suppress small RNA mediated silencing (Qiao et al., 2013), thus possibility exists that SRGE conferred viral immunity may be broken in mixed infection with Oomycete pathogen. Interestingly it was recently reported that miRNA can be exported to fungal cells and inhibit pathogen gene expression thus confer resistance (Zhang et al., 2016). Since Oomycete and fungi are both eukaryotes where silencing operates, thus a possible solution to breaking down SRGE by Oomycete (and possibly fungi as well) is to target it together with viruses by SRGE.

Small RNA mediated silencing is also affected by abiotic stress, such as low and high temperature, drought and salt stress, which are often encountered in crop cultivation. Investigation of molecular mechanism by which those abiotic stresses manipulate silencing pathway, will provide solution to proper compensation strategy for SRGE application in those stress conditions.

It was reported early that small RNA mediated silencing in non-cell autonomous and silencing signal is capable of both cell-to-cell and phloem dependent long distance movement (Palauqui et al., 1997; Voinnet and Baulcombe, 1997). In modern horticulture, grafted seedlings were widely used in vegetable and fruit tree cultivation in which crop scions are grafted onto rootstock of related species. Grafted crops usually perform better compared to their self-rooted counterpart in terms of nutrient efficiency, abiotic stress tolerance and resistance to soil born disease. It is worthwhile to explore the possibility to generate viral resistant rootstock with SRGE to provide protection for different crop scions. This way can save the effort to introduce resistance trait to every commercial varieties or develop transformation system for them, which are time consuming and sometimes not possible for certain species. Though, AMIR mediated resistance failed to cross graft union (Zhang et al., 2011a), many other types of small RNAs remain to be tested for this potential and grafting methods can be further optimized.

Finally, plant genomes encode multiple DCL genes capable of generating miRNA and siRNAs in many ways. Fully dissection of the small RNA biogenesis mechanisms mediated by those different DCL proteins, can help design silencing constructs expressing as many as possible small RNAs, which holds the key for success of SRGE application in crop protection.

## Author Contributions

AK and FL conducted literature research and analyzed the data. FL and AK wrote the manuscript. QZ contributed **Figure 1**. MY contributed in literature research.

## Conflict of Interest Statement

The authors declare that the research was conducted in the absence of any commercial or financial relationships that could be construed as a potential conflict of interest.

## References

[B1] Abel P. P. Nelson R. S. De B. Hoffmann N. Rogers S. G. Fraley R. T. (1986). Delay of disease development in transgenic plants that express the tobacco mosaic virus coat protein gene. *Science* 232 738–743. 10.1126/science.3457472 3457472

[B2] Alazem M. Lin N. S. (2015). Roles of plant hormones in the regulation of host-virus interactions. *Mol. Plant Pathol.* 16 529–540. 10.1111/mpp.12204 25220680 PMC6638471

[B3] Antignus Y. Vunsh R. Lachman O. Pearlsman M. Maslenin L. Hananya U. (2004). Truncated rep gene originated from tomato yellow leaf curl virus-Israel [Mild] confers strain-specific resistance in transgenic tomato. *Ann. Appl. Biol.* 144 39–44. 10.1111/j.1744-7348.2004.tb00314.x

[B4] Aragao F. J. L. Nogueira E. O. P. L. Tinoco M. L. P. Faria J. C. (2013). Molecular characterization of the first commercial transgenic common bean immune to the Bean golden mosaic virus. *J. Biotechnol.* 166 42–50. 10.1016/j.jbiotec.2013.04.009 23639387

[B5] Arif M. Azhar U. Arshad M. Zafar Y. Mansoor S. Asad S. (2012). Engineering broad-spectrum resistance against RNA viruses in potato. *Transgenic Res.* 21 303–311. 10.1007/s11248-011-9533-7 21701953

[B6] Arif M. Thomas P. E. Crosslin J. M. Brown C. R. (2009). Development of molecular resistance in potato against potato leaf roll virus and potato virus y through agrobacterium-mediated double transgenesis. *Pak. J. Bot.* 41 945–954.

[B7] Batuman O. Mawassi M. Bar-Joseph M. (2006). Transgenes consisting of a dsRNA of an RNAi suppressor plus the 3 ’ UTR provide resistance to *Citrus tristeza* virus sequences in *Nicotiana benthamiana* but not in citrus. *Virus Genes* 33 319–327. 16991003 10.1007/s11262-006-0071-y

[B8] Bau H. J. Cheng Y. I. H. Yu T. A. Yang J. S. Yeh S. D. (2003). Broad-spectrum resistance to different geographic strains of *Papaya ringspot* virus in coat protein gene transgenic papaya. *Phytopathology* 93 112–120. 10.1094/PHYTO.2003.93.1.112 18944164

[B9] Baulcombe D. (2004). RNA silencing in plants. *Nature* 431 356–363. 10.1038/nature02874 15372043

[B10] Baulcombe D. C. (1996). Mechanisms of pathogen-derived resistance to viruses in transgenic plants. *Plant Cell* 8 1833–1844. 10.1105/tpc.8.10.1833 12239365 PMC161318

[B11] Baulcombe D. C. (2015). VIGS, HIGS and FIGS: small RNA silencing in the interactions of viruses or filamentous organisms with their plant hosts. *Curr. Opin. Plant Biol.* 26 141–146. 10.1016/j.pbi.2015.06.007 26247121

[B12] Bouche N. Lauressergues D. Gasciolli V. Vaucheret H. (2006). An antagonistic function for *Arabidopsis* DCL2 in development and a new function for DCL4 in generating viral siRNAs. *EMBO J.* 25 3347–3356. 10.1038/sj.emboj.7601217 16810317 PMC1523179

[B13] Bucher E. Lohuis D. van Poppel P. M. Geerts-Dimitriadou C. Goldbach R. Prins M. (2006). Multiple virus resistance at a high frequency using a single transgene construct. *J. Gen. Virol.* 87 3697–3701. 10.1099/vir.0.82276-0 17098987

[B14] Burgyan J. Havelda Z. (2011). Viral suppressors of RNA silencing. *Trends Plant Sci.* 16 265–272. 10.1016/j.tplants.2011.02.010 21439890

[B15] Cao X. L. Lu Y. G. Di D. P. Zhang Z. Y. Liu H. Tian L. Z. (2013). Enhanced virus resistance in transgenic maize expressing a dsRNA-Specific endoribonuclease gene from *E. coli*. *PLoS ONE* 8:e60829 10.1371/journal.pone.0060829 PMC3621894 23593318

[B16] Carbonell A. Carrington J. C. (2015). Antiviral roles of plant ARGONAUTES. *Curr. Opin. Plant Biol.* 27 111–117. 10.1016/j.pbi.2015.06.013 26190744 PMC4618181

[B17] Chellappan P. Masona M. V. Vanitharani R. Taylor N. J. Fauquet C. M. (2004). Broad spectrum resistance to ssDNA viruses associated with transgene-induced gene silencing in cassava. *Plant Mol. Biol.* 56 601–611. 10.1007/s11103-004-0147-9 15630623

[B18] Chen L. Y. Cheng X. F. Cai J. Y. Zhan L. L. Wu X. X. Liu Q. (2016). Multiple virus resistance using artificial trans-acting siRNAs. *J. Virol. Methods* 228 16–20. 10.1016/j.jviromet.2015.11.004 26562057

[B19] Chen X. (2012). Small RNAs in development–insights from plants. *Curr. Opin. Genet. Dev.* 22 361–367. 10.1016/j.gde.2012.04.004 22578318 PMC3419802

[B20] Cruz L. F. Rupp J. L. S. Trick H. N. Fellers J. P. (2014). Stable resistance to Wheat streak mosaic virus in wheat mediated by RNAi. *Crop Sci.* 50 665–672. 10.1007/s11627-014-9634-0

[B21] Csorba T. Kontra L. Burgyan J. (2015). viral silencing suppressors: tools forged to fine-tune host-pathogen coexistence. *Virology* 47 85–103. 10.1016/j.virol.2015.02.028 25766638

[B22] Czech B. Hannon G. J. (2011). Small RNA sorting: matchmaking for Argonautes. *Nat. Rev. Genet.* 12 19–31. 10.1038/nrg2916 21116305 PMC3703915

[B23] Czosnek H. Laterrot H. (1997). A worldwide survey of tomato yellow leaf curl viruses. *Arch. Virol.* 142 1391–1406. 10.1007/s007050050168 9267451

[B24] Dai S. Beachy R. N. (2009). Genetic engineering of rice to resist rice tungro disease. *In Vitro Cell. Dev. Biol. Plant* 45 517–524. 10.1007/s11627-009-9241-7

[B25] Deleris A. Gallego-Bartolome J. Bao J. Kasschau K. D. Carrington J. C. Voinnet O. (2006). Hierarchical action and inhibition of plant Dicer-like proteins in antiviral defense. *Science* 313 68–71. 10.1126/science.1128214 16741077

[B26] Diaz-Pendon J. A. Truniger V. Nieto C. Garcia-Mas J. Bendahmane A. Aranda M. A. (2004). Advances in understanding recessive resistance to plant viruses. *Mol. Plant Pathol.* 5 223–233. 10.1111/j.1364-3703.2004.00223.x 20565612

[B27] Ding S. W. (2000). RNA silencing. *Curr. Opin. Biotechnol.* 11 152–156. 10.1016/S0958-1669(00)00074-4 10753772

[B28] Ding S. W. (2010). RNA-based antiviral immunity. *Nat. Rev. Immunol.* 10 632–644. 10.1038/nri2824 20706278

[B29] Ding S. W. Lu R. (2011). Virus-derived siRNAs and piRNAs in immunity and pathogenesis. *Curr. Opin. Virol.* 1 533–544. 10.1016/j.coviro.2011.10.028 22180767 PMC3237678

[B30] Ding S. W. Voinnet O. (2007). Antiviral immunity directed by small RNAs. *Cell* 130 413–426. 10.1016/j.cell.2007.07.039 17693253 PMC2703654

[B31] Doreste V. Ramos P. L. Enriquez G. A. Rodriguez R. Peral R. Pujol M. (2002). Transgenic potato plants expressing the potato virus X (PVX) coat protein gene developed resistance to the viral infection. *Phytoparasitica* 30 177–185. 10.1007/BF02979700

[B32] Duan C. G. Wang C. H. Fang R. X. Guo H. S. (2008). Artificial MicroRNAs highly accessible to targets confer efficient virus resistance in plants. *J. Virol.* 82 11084–11095. 10.1128/JVI.01377-08 18768978 PMC2573272

[B33] Ehrenfeld N. Romano E. Serrano C. Arce-Johnson P. (2004). Replicase mediated resistance against potato leafroll virus in potato desiree plants. *Biol. Res.* 37 71–82. 10.4067/S0716-97602004000100008 15174307

[B34] Elayabalan S. Kalaiponmani K. Subramaniam S. Selvarajan R. Panchanathan R. Muthuvelayoutham R. (2013). Development of agrobacterium-mediated transformation of highly valued hill banana cultivar Virupakshi (AAB) for resistance to BBTV disease. *World J Microbiol. Biotechnol.* 29 589–596. 10.1007/s11274-012-1214-z 23184576

[B35] Fahim M. Ayala-Navarrete L. Millar A. A. Larkin P. J. (2010). Hairpin RNA derived from viral NIa gene confers immunity to wheat streak mosaic virus infection in transgenic wheat plants. *Plant Biotechnol. J.* 8 821–834. 10.1111/j.1467-7652.2010.00513.x 20374525

[B36] Fahim M. Millar A. A. Wood C. C. Larkin P. J. (2012). Resistance to Wheat streak mosaic virus generated by expression of an artificial polycistronic microRNA in wheat. *Plant Biotechnol. J.* 10 150–163. 10.1111/j.1467-7652.2011.00647.x 21895944

[B37] Faria J. C. Albino M. M. C. Dias B. B. A. Cancado L. J. da Cunha N. B. Silva L. D. (2006). Partial resistance to Bean golden mosaic virus in a transgenic common bean (*Phaseolus vulgar* L.) line expressing a mutated rep gene. *Plant Sci.* 171 565–571. 10.1016/j.plantsci.2006.06.010

[B38] Faria J. C. Valdisser P. A. M. R. Nogueira E. O. P. L. Aragao F. J. L. (2014). RNAi-based Bean golden mosaic virus-resistant common bean (Embrapa 5.1) shows simple inheritance for both transgene and disease resistance. *Plant Breed.* 133 649–653. 10.1111/pbr.12189

[B39] Ferreira S. A. Pitz K. Y. Manshardt R. Zee F. Fitch M. Gonsalves D. (2002). Virus coat protein Transgenic papaya provides practical control of *Papaya ringspot* virus in Hawaii. *Plant Dis.* 86 101–105. 10.1094/PDIS.2002.86.2.101 30823304

[B40] Fire A. Xu S. Montgomery M. K. Kostas S. A. Driver S. E. Mello C. C. (1998). Potent and specific genetic interference by double-stranded RNA in *Caenorhabditis elegans*. *Nature* 391 806–811. 10.1038/35888 9486653

[B41] Fitch M. M. M. Manshardt R. M. Gonsalves D. Slightom J. L. Sanford J. C. (1992). Virus resistant papaya plants derived from tissues bombarded with the coat protein gene of *Papaya ringspot* virus. *Nat. Biotechnol.* 10 1466–1472. 10.1038/nbt1192-1466

[B42] Fu Z. Q. Dong X. (2013). Systemic acquired resistance: turning local infection into global defense. *Annu. Rev. Plant Biol.* 64 839–863. 10.1146/annurev-arplant-042811-105606 23373699

[B43] Fuchs M. Gonsalves D. (2007). Safety of virus-resistant transgenic plants two decades after their introduction: lessons from realistic field risk assessment studies. *Annu. Rev. Phytopathol.* 45 173–202. 10.1146/annurev.phyto.45.062806.094434 17408355

[B44] Fuentes A. Ramos P. L. Fiallo E. Callard D. Sanchez Y. Peral R. (2006). Intron-hairpin RNA derived from replication associated protein C1 gene confers immunity to tomato yellow leaf curl virus infection in transgenic tomato plants. *Transgenic Res.* 15 291–304. 10.1007/s11248-005-5238-0 16779645

[B45] Gal-On A. Wolf D. Antignus Y. Patlis L. Ryu K. H. Min B. E. (2005). Transgenic cucumbers harboring the 54-kDa putative gene of Cucumber fruit mottle mosaic tobamovirus are highly resistant to viral infection and protect non-transgenic scions from soil infection. *Transgenic Res.* 14 81–93. 10.1007/s11248-004-3802-7 15865051

[B46] Gao L. Ding X. Li K. Liao W. Zhong Y. Ren R. (2015). Characterization of Soybean mosaic virus resistance derived from inverted repeat-SMV-HC-Pro genes in multiple soybean cultivars. *Theor. Appl. Genet.* 128 1489–1505. 10.1007/s00122-015-2522-0 25930057

[B47] Gielen J. J. de Haan P. Kool A. J. Peters D. Van Grinsven M. Q. Goldbach R. W. (1991). Engineered resistance to tomato spotted wilt virus, a negative–strand RNA virus. *Nat. Biotechnol.* 9 1363–1367. 10.1038/nbt1291-1363

[B48] Gonsalves D. (2006). Transgenic papaya: development, release, impact and challenges. *Adv. Virus Res.* 67 317–354. 10.1016/S0065-3527(06)67009-7 17027684

[B49] Guo H. S. Cervera M. T. Garcia J. A. (1998). Plum pox potyvirus resistance associated to transgene silencing that can be stabilized after different number of plant generations. *Gene* 206 263–272. 10.1016/S0378-1119(97)00595-7 9469941

[B50] Higgins C. M. Hall R. M. Mitter N. Cruickshank A. Dietzgen R. G. (2004). Peanut stripe potyvirus resistance in peanut (*Arachis hypogaea* L.) plants carrying viral coat protein gene sequences. *Transgenic Res.* 13 59–67. 10.1023/B:TRAG.0000017166.29458.74 15070076

[B51] Hily J. M. Ravelonandro M. Damsteegt V. Bassett C. Petri C. Liu Z. (2007). Plum pox virus coat protein gene Intron-hairpin-RNA (ihpRNA) constructs provide resistance to plum pox virus in *Nicotiana benthamiana* and *Prunus domestica*. *J. Am. Soc. Hortic. Sci.* 132 850–858.

[B52] Jiang L. Wei C. Li Y. (2012). Viral suppression of RNA silencing. *Sci. China Life Sci.* 55 109–118. 10.1007/s11427-012-4279-x 22415681

[B53] Jones J. D. Dangl J. L. (2006). The plant immune system. *Nature* 444 323–329. 10.1038/nature05286 17108957

[B54] Kalantidis K. Psaradakis S. Tabler M. Tsagris M. (2002). The occurrence of CMV-specific short Rnas in transgenic tobacco expressing virus-derived double-stranded RNA is indicative of resistance to the virus. *Mol. Plant Microbe Interact.* 15 826–833. 10.1094/MPMI.2002.15.8.826 12182340

[B55] King A. Adams M. Carstens E. Lefkowitz E. (2012). *Virus Taxonomy: Ninth Report of the International Committee on Taxonomy of Viruses*. Amsterdam: Elsevier Academic Press.

[B56] Kojima R. Lapierre H. (1988). “Potato leafroll virus,” in *European Handbook of Plant Diseases*, eds Smith I. M. Dunez V. Philips D. H. Leliot R. A. Archer S. A. (Oxford: Blackwell Scientific Publications), 23–24.

[B57] Krubphachaya P. Juricek M. Kertbundit S. (2007). Induction of RNA-mediated resistance to *Papaya ringspot* virus type W. *J. Biochem. Mol. Biol.* 40 404–411. 17562292 10.5483/bmbrep.2007.40.3.404

[B58] Kung Y. J. Bau H. J. Wu Y. L. Huang C. H. Chen T. M. Yeh S. D. (2009). Generation of transgenic Papaya with double resistance to *Papaya ringspot* virus and Papaya leaf-distortion mosaic virus. *Phytopathology* 99 1312–1320. 10.1094/PHYTO-99-11-1312 19821736

[B59] Kung Y. J. You B. J. Raja J. A. J. Chen K. C. Huang C. H. Bau H. J. (2015). nucleotide sequence-homology-independent breakdown of transgenic resistance by more virulent virus strains and a potential solution. *Sci. Rep.* 5:9804 10.1038/srep09804 PMC5386206 25913508

[B60] Legg J. P. Jeremiah S. C. Obiero H. M. Maruthi M. N. Ndyetabula I. Okao-Okuja G. (2011). Comparing the regional epidemiology of the cassava mosaic and cassava brown streak virus pandemics in Africa. *Virus Res.* 159 161–170. 10.1016/j.virusres.2011.04.018 21549776

[B61] Li F. Ding S. W. (2006). Virus counterdefense: diverse strategies for evading the RNA-silencing immunity. *Annu. Rev. Microbiol.* 60 503–531. 10.1146/annurev.micro.60.080805.142205 16768647 PMC2693410

[B62] Lin C. Y. Ku H. M. Chiang Y. H. Ho H. Y. Yu T. A. Jan F. J. (2012). Development of transgenic watermelon resistant to Cucumber mosaic virus and Watermelon mosaic virus by using a single chimeric transgene construct. *Transgenic Res.* 21 983–993. 10.1007/s11248-011-9585-8 22203520

[B63] Lindbo J. A. Silva-Rosales L. Proebsting W. M. Dougherty W. G. (1993). Induction of a highly specific antiviral state in transgenic plants: implications for regulation of gene expression and virus resistance. *Plant Cell* 5 1749–1759. 10.2307/3869691 12271055 PMC160401

[B64] Liu Z. R. Scorza R. Hily J. M. Scott S. W. James D. (2007). Engineering resistance to multiple Prunus fruit viruses through expression of chimeric hairpins. *J. Am. Soc. Hortic. Sci.* 132 407–414.

[B65] Ma Z. L. Yang H. Y. Wang R. Tien P. (2004). Construct hairpin RNA to fight against rice dwarf virus. *Acta Bot. Sin.* 46 332–336.

[B66] Masmoudi K. Yacoubi I. Hassairi A. Elarbi L. N. Ellouz R. (2002). Tobacco plants transformed with an untranslatable form of the coat protein gene of the Potato virus Y are resistant to viral infection. *Eur. J. Plant Pathol.* 108 285–292. 10.1023/A:1015656017326

[B67] Mehta R. Radhakrishnan T. Kumar A. Yadav R. Dobaria J. R. Thirumalaisamy P. P. (2013). Coat protein-mediated transgenic resistance of peanut (*Arachis hypogaea* L.) to peanut stem necrosis disease through Agrobacterium-mediated genetic transformation. *Indian J. Virol.* 24 205–213. 10.1007/s13337-013-0157-9 24426277 PMC3784911

[B68] Mlotshwa S. Pruss G. J. Vance V. (2008). Small RNAs in viral infection and host defense. *Trends Plant Sci.* 13 375–382. 10.1016/j.tplants.2008.04.009 18550416

[B69] Mourrain P. Beclin C. Elmayan T. Feuerbach F. Godon C. Morel J. B. (2000). *Arabidopsis* SGS2 and SGS3 genes are required for posttranscriptional gene silencing and natural virus resistance. *Cell* 101 533–542. 10.1016/S0092-8674(00)80863-6 10850495

[B70] Nicaise V. (2014). Crop immunity against viruses: outcomes and future challenges. *Front. Plant Sci.* 5:660 10.3389/fpls.2014.00660 PMC4240047 25484888

[B71] Niu Q. W. Lin S. S. Reyes J. L. Chen K. C. Wu H. W. Yeh S. D. (2006). Expression of artificial microRNAs in transgenic *Arabidopsis thaliana* confers virus resistance. *Nat. Biotechnol.* 24 1420–1428. 10.1038/nbt1255 17057702

[B72] Nunome T. Fukumoto F. Terami F. Hanada K. Hirai M. (2002). Development of breeding materials of transgenic tomato plants with a truncated replicase gene of cucumber mosaic virus for resistance to the virus. *Breed. Sci.* 52 219–223. 10.1270/jsbbs.52.219

[B73] Palauqui J. C. Elmayan T. Pollien J. M. Vaucheret H. (1997). Systemic acquired silencing: transgene-specific post-transcriptional silencing is transmitted by grafting from silenced stocks to non-silenced scions. *EMBO J.* 16 4738–4745. 10.1093/emboj/16.15.4738 9303318 PMC1170100

[B74] Palukaitis P. (2011). The Road to RNA silencing is paved with plant-virus interactions. *Plant Pathol. J.* 27 197–206. 10.5423/PPJ.2011.27.3.197

[B75] Pang S. Z. Jan F. J. Tricoli D. M. Russell P. F. Carney K. J. Hu J. S. (2000). Resistance to squash mosaic comovirus in transgenic squash plants expressing its coat protein genes. *Mol. Breed.* 6 87–93. 10.1023/A:1009619230918

[B76] Prins M. de Haan P. Luyten R. van Veller M. van Grinsven M. Q. Goldbach R. (1995). Broad resistance to tospoviruses in transgenic tobacco plants expressing three tospoviral nucleoprotein gene sequences. *Mol. Plant Microbe Interact.* 8 85–91. 10.1094/MPMI-8-0085 7772807

[B77] Prins M. Kikkert M. Ismayadi C. de Graauw W. de Haan P. Goldbach R. (1997). Characterization of RNA-mediated resistance to tomato spotted wilt virus in transgenic tobacco plants expressing NS(M) gene sequences. *Plant Mol. Biol.* 33 235–243. 10.1023/A:1005729808191 9037142

[B78] Prins M. Resende Rde O. Anker C. van Schepen A. de Haan P. Goldbach R. (1996). Engineered RNA-mediated resistance to tomato spotted wilt virus is sequence specific. *Mol. Plant Microbe Interact.* 9 416–418. 10.1094/MPMI-9-0416 8672819

[B79] Qiao Y. Liu L. Xiong Q. Flores C. Wong J. Shi J. (2013). Oomycete pathogens encode RNA silencing suppressors. *Nat. Genet.* 45 330–333. 10.1038/ng.2525 23377181 PMC4049077

[B80] Qu J. Ye J. Fang R. (2007). Artificial microRNA-mediated virus resistance in plants. *J. Virol.* 81 6690–6699. 10.1128/JVI.02457-06 17344304 PMC1900123

[B81] Ravelonandro M. Scorza R. Michel H. J. Briard P. (2014). The efficiency of RNA interference for conferring stable resistance to plum pox virus. *Plant Cell Tissue Organ Cult.* 118 347–356. 10.1007/s11240-014-0487-3

[B82] Reyes C. A. De Francesco A. Pena E. J. Costa N. Plata M. I. Sendin L. (2011). Resistance to *Citrus psorosis* virus in transgenic sweet orange plants is triggered by coat protein-RNA silencing. *J. Biotechnol.* 151 151–158. 10.1016/j.jbiotec.2010.11.007 21084056

[B83] Roger H. (2014). *Plant Virology*, 5th Edn Cambridge, MA: Academic Press.

[B84] Romay G. Lecoq H. Desbiez C. (2014). CUCURBIT CROPS AND THEIR VIRAL DISEASES IN LATIN AMERICA AND THE CARIBBEAN ISLANDS: A REVIEW. *J. Plant Pathol.* 96 227–242.

[B85] Roy S. Banerjee A. Tarafdar J. Senapati B. K. Dasgupta I. (2012). Transfer of transgenes for resistance to rice tungro into high-yielding rice cultivars through gene-based marker-assisted selection. *J. Agric. Sci.* 150 610–618. 10.1017/S0021859611000827

[B86] Sanford J. C. Johnston S. A. (1985). The concept of parasite-derived resistance—Deriving resistance genes from the parasite’s own genome. *J. Theor. Biol.* 113 395–405. 10.1016/S0022-5193(85)80234-4

[B87] Sasaya T. Nakazono-Nagaoka E. Saika H. Aoki H. Hiraguri A. Netsu O. (2014). Transgenic strategies to confer resistance against viruses in rice plants. *Front. Microbiol.* 4:409 10.3389/fmicb.2013.00409 PMC3888933 24454308

[B88] Savenkov E. I. Valkonen J. P. (2001). Coat protein gene-mediated resistance to Potato virus A in transgenic plants is suppressed following infection with another potyvirus. *J. Gen. Virol.* 82 2275–2278. 10.1099/0022-1317-82-9-2275 11514739

[B89] Scorza R. Callahan A. Levy L. Damsteegt V. Webb K. Ravelonandro M. (2001). Post-transcriptional gene silencing in plum pox virus resistant transgenic European plum containing the plum pox potyvirus coat protein gene. *Transgenic Res.* 10 201–209. 10.1023/A:1016644823203 11437277

[B90] Shekhawat U. K. S. Ganapathi T. R. Hadapad A. B. (2012). Transgenic banana plants expressing small interfering RNAs targeted against viral replication initiation gene display high-level resistance to banana bunchy top virus infection. *J. Gen. Virol.* 93 1804–1813. 10.1099/vir.0.041871-0 22552945

[B91] Shepherd D. N. Mangwende T. Martin D. P. Bezuidenhout M. Thomson J. A. Rybicki E. P. (2007). Inhibition of maize streak virus (MSV) replication by transient and transgenic expression of MSV replication-associated protein mutants. *J. Gen. Virol.* 88 325–336. 10.1099/vir.0.82338-0 17170465

[B92] Sherwood J. L. German T. L. Moyer J. W. Ullman D. E. (2003). *Tomato Spotted wilt. The Plant Health Instructor*. Available at: http://www.apsnet.org/edcenter/intropp/lessons/viruses/Pages/TomatoSpottedWilt.aspx

[B93] Shimizu T. Nakazono-Nagaoka E. Uehara-Ichiki T. Sasaya T. Omura T. (2011). Targeting specific genes for RNA interference is crucial to the development of strong resistance to rice stripe virus. *Plant Biotechnol. J.* 9 503–512. 10.1111/j.1467-7652.2010.00571.x 21040387

[B94] Shin R. Han J. H. Lee G. J. Peak K. H. (2002). The potential use of a viral coat protein gene as a transgene screening marker and multiple virus resistance of pepper plants coexpressing coat proteins of cucumber mosaic virus and tomato mosaic virus. *Transgenic Res.* 11 215–219. 10.1023/A:1015200622716 12054354

[B95] Simon-Mateo C. Garcia J. A. (2011). Antiviral strategies in plants based on RNA silencing. *Biochim. Biophys. Acta* 1809 722–731. 10.1016/j.bbagrm.2011.05.011 21652000

[B96] Simon-Mateo C. Lopez-Moya J. J. Guo H. S. Gonzalez E. Garcia J. A. (2003). Suppressor activity of potyviral and cucumoviral infections in potyvirus-induced transgene silencing. *J. Gen. Virol.* 84 2877–2883. 10.1099/vir.0.19263-0 13679623

[B97] Sivamani E. Brey C. Dyer W. E. Talbert L. E. Qu R. (2000). Resistance to wheat streak mosaic virus in transgenic wheat expressing the viral replicase (NIb) gene. *Mol. Breed.* 6 469–477. 10.1023/A:1026576124482

[B98] Sivamani E. Brey C. W. Talbert L. E. Young M. A. Dyer W. E. Kaniewski W. K. (2002). Resistance to wheat streak mosaic virus in transgenic wheat engineered with the viral coat protein gene. *Transgenic Res.* 11 31–41. 10.1023/A:1013944011049 11874101

[B99] Soler N. Plomer M. Fagoaga C. Moreno P. Navarro L. Flores R. (2012). Transformation of Mexican lime with an intron-hairpin construct expressing untranslatable versions of the genes coding for the three silencing suppressors of *Citrus tristeza* virus confers complete resistance to the virus. *Plant Biotechnol. J.* 10 597–608. 10.1111/j.1467-7652.2012.00691.x 22405601

[B100] Taylor N. Chavarriaga P. Raemakers K. Siritunga D. Zhang P. (2004). Development and application of transgenic technologies in cassava. *Plant Mol. Biol.* 56 671–688. 10.1007/s11103-004-4872-x 15630627

[B101] Truniger V. Aranda M. A. (2009). Recessive resistance to plant viruses. *Adv. Virus Res.* 75 119–159. 10.1016/S0065-3527(09)07504-6 20109665

[B102] Tyagi H. Rajasubramaniam S. Rajam M. V. Dasgupta I. (2008). RNA-interference in rice against Rice tungro bacilliform virus results in its decreased accumulation in inoculated rice plants. *Transgenic Res.* 17 897–904. 10.1007/s11248-008-9174-7 18306054 PMC2522301

[B103] Valarmathi P. Kumar G. Robin S. Manonmani S. Dasgupta I. Rabindran R. (2016). Evaluation of virus resistance and agronomic performance of rice cultivar ASD 16 after transfer of transgene against Rice tungro bacilliform virus by backcross breeding. *Virus Genes* 52 521–529. 10.1007/s11262-016-1318-x 26983604

[B104] Vance V. Vaucheret H. (2001). RNA silencing in plants–defense and counterdefense. *Science* 292 2277–2280. 10.1126/science.1061334 11423650

[B105] Vanderschuren H. Akbergenov R. Pooggin M. M. Hohn T. Gruissem W. Zhang P. (2007). Transgenic cassava resistance to African cassava mosaic virus is enhanced by viral DNA-A bidirectional promoter-derived siRNAs. *Plant Mol. Biol.* 64 549–557. 10.1007/s11103-007-9175-6 17492253

[B106] Vlot A. C. Dempsey D. A. Klessig D. F. (2009). Salicylic Acid, a multifaceted hormone to combat disease. *Annu. Rev. Phytopathol.* 47 177–206. 10.1146/annurev.phyto.050908.135202 19400653

[B107] Voinnet O. (2005). Induction and suppression of RNA silencing: insights from viral infections. *Nat. Rev. Genet.* 6 206–220. 10.1038/nrg1555 15703763

[B108] Voinnet O. Baulcombe D. C. (1997). Systemic signalling in gene silencing. *Nature* 389 553 10.1038/39215 9335491

[B109] Vu T. V. Choudhury N. R. Mukherjee S. K. (2013). Transgenic tomato plants expressing artificial microRNAs for silencing the pre-coat and coat proteins of a begomovirus, Tomato leaf curl New Delhi virus, show tolerance to virus infection. *Virus Res.* 172 35–45. 10.1016/j.virusres.2012.12.008 23276684

[B110] Wang A. Krishnaswamy S. (2012). Eukaryotic translation initiation factor 4E-mediated recessive resistance to plant viruses and its utility in crop improvement. *Mol. Plant Pathol.* 13 795–803. 10.1111/j.1364-3703.2012.00791.x 22379950 PMC6638641

[B111] Wang F. Li W. Zhu J. Fan F. Wang J. Zhong W. (2016). Hairpin RNA targeting multiple viral genes confers strong resistance to rice black-streaked dwarf virus. *Int. J. Mol. Sci.* 17:E705 10.3390/ijms17050705 PMC4881527 27187354

[B112] Wang X. Eggenberger A. L. Nutter F. W. Hill J. H. (2001). Pathogen-derived transgenic resistance to soybean mosaic virus in soybean. *Mol. Breed.* 8 119–127. 10.1007/s00122-015-2522-0

[B113] Wassenegger M. Krczal G. (2006). Nomenclature and functions of RNA-directed RNA polymerases. *Trends Plant Sci.* 11 142–151. 10.1016/j.tplants.2006.01.003 16473542

[B114] Waterhouse P. M. Graham M. W. Wang M. B. (1998). Virus resistance and gene silencing in plants can be induced by simultaneous expression of sense and antisense RNA. *Proc. Natl. Acad. Sci. U.S.A.* 95 13959–13964. 10.1073/pnas.95.23.13959 9811908 PMC24986

[B115] Wittner A. Palkovics L. Balazs E. (1998). *Nicotiana benthamiana* plants transformed with the plum pox virus helicase gene are resistant to virus infection. *Virus Res.* 53 97–103. 10.1016/S0168-1702(97)00133-0 9617773

[B116] Wu H. W. Yu T. A. Raja J. A. J. Christopher S. J. Wang S. L. Yeh S. D. (2010). Double–virus resistance of transgenic oriental melon conferred by untranslatable chimeric construct carrying partial coat protein genes of two viruses. *Plant Dis.* 94 1341–1347. 10.1094/PDIS-11-09-0742 30743648

[B117] Zanek M. C. Reyes C. A. Cervera M. Pena E. J. Velazquez K. Costa N. (2008). Genetic transformation of sweet orange with the coat protein gene of *Citrus psorosis* virus and evaluation of resistance against the virus. *Plant Cell Rep.* 27 57–66. 10.1007/s00299-007-0422-8 17712560

[B118] Zhang C. Wu Z. Li Y. Wu J. (2015). Biogenesis, function, and applications of virus-derived small RNAs in plants. *Front. Microbiol.* 6:1237 10.3389/fmicb.2015.01237 PMC4637412 26617580

[B119] Zhang T. Zhao Y. L. Zhao J. H. Wang S. Jin Y. Chen Z. Q. (2016). Cotton plants export microRNAs to inhibit virulence gene expression in a fungal pathogen. *Nat. Plants* 2:16153 10.1038/nplants.2016.153 27668926

[B120] Zhang X. Li H. Zhang J. Zhang C. Gong P. Ziaf K. (2011a). Expression of artificial microRNAs in tomato confers efficient and stable virus resistance in a cell-autonomous manner. *Transgenic Res.* 20 569–581. 10.1007/s11248-010-9440-3 20835923

[B121] Zhang X. Sato S. Ye X. Dorrance A. E. Morris T. J. Clemente T. E. (2011b). Robust RNAi-based resistance to mixed infection of three viruses in soybean plants expressing separate short hairpins from a single transgene. *Phytopathology* 101 1264–1269. 10.1094/PHYTO-02-11-0056 21999157

[B122] Zhang Z. Y. Yang L. Zhou S. F. Wang H. G. Li W. C. Fu F. L. (2011). Improvement of resistance to maize dwarf mosaic virus mediated by transgenic RNA interference. *J. Biotechnol.* 153 181–187. 10.1016/j.jbiotec.2011.03.019 21504770

[B123] Zhang Z. Y. Fu F. L. Gou L. Wang H. G. Li W. C. (2010). RNA Interference-Based Transgenic maize resistant to maize dwarf mosaic virus. *J. Plant Biol.* 53 297–305. 10.1016/j.jbiotec.2011.03.019

[B124] Zhang Z. Y. Wang Y. G. Shen X. J. Li L. Zhou S. F. Li W. C. (2013). RNA interference-mediated resistance to maize dwarf mosaic virus. *Plant Cell Tissue Organ Cult.* 113 571–578. 10.1007/s11240-013-0289-z

[B125] Zitter T. A. Murphy J. F. (2009). *Cucumber Mosaic. The Plant Health Instructor*. Available at: http://www.apsnet.org/edcenter/intropp/lessons/viruses/Pages/Cucumbermosaic.aspx

