# Supplementary material for: Small RNA Based Genetic Engineering for Plant Viral Resistance: Application in Crop Protection
Source: Front Microbiol. 2017 Jan 23;8:43. doi: 10.3389/fmicb.2017.00043 (PMC5253543; doi:10.3389/fmicb.2017.00043)
Supplement: Supplementary file 1 [file Table_1.DOCX]

Table S1 List of small RNA based genetic engineering for viral resistance in crops and tobacco plants.

| common name | *latin name* | *plant family* | virus | virus name (full) | virus genera | genome type | viral target | mechanism | phenotype | references |
| --- | --- | --- | --- | --- | --- | --- | --- | --- | --- | --- |
| **Sweet Orange** | *Citrus sinensis* | *RUTACEAE* | CTV | Citrus tristeza virus | Closterovirus | + SS RNA | 3^/^ end,3^/^ end+p23 gene | S-PTGS | Moderate | (Olivares-Fuster et al., 2003) |
| **Key Lime** | *Citrus aurantifolia* | *RUTACEAE* | CTV | Citrus tristeza virus | Closterovirus | + SS RNA | P25 CP | CPMR | Moderate | (Dominguez et al., 2002) |
| **Grape Fruit** | *Citrus paradise* | *RUTACEAE* | CTV | Citrus tristeza virus | Closterovirus | + SS RNA | CP, p27, 3END-S, RdRp | S-PTGS | Moderate | (Febres et al., 2008) |
| **Grape Fruit** | *Citrus paradise,* | *RUTACEAE* | CTV | Citrus tristeza virus | Closterovirus | + SS RNA | p25 gene | CPMR | Lower virus titre | (Loeza-Kuk et al., 2011) |
| **Mexican Lime** | *Citrus aurantifolia* | *RUTACEAE* | CTV | Citrus tristeza virus | Closterovirus | + SS RNA | p26 gene | CPMR | Lower virus titre | (Loeza-Kuk et al., 2011) |
| **Mexican Lime** | *Citrus aurantifolia* | *RUTACEAE* | CTV | Citrus tristeza virus | Closterovirus | + SS RNA | p25, p20 and p23 + 3^/^ end | Hp-PTGS | Complete resistance | (Soler et al., 2012) |
| **Alemow** | *Citrus macrophylla* | *RUTACEAE* | CTV | Citrus tristeza virus | Closterovirus | + SS RNA | P23+4'UTR | Hp-PTGS | No resistance | (Batuman et al., 2006) |
| **Hardy Orange** | *Poncirus trifoliate* | *RUTACEAE* | CiMV | Citrus mosaic virus | Nepovirus | +SS RNA | capsid polyprotein gene | S-PTGS | Lower infection | (Iwanami et al., 2004) |
| **Sweet Orange** | *Citrus sinensis* | *RUTACEAE* | CPsV | Citrus psorosis virus | Ophiovirus | - SS RNA | CP gene | S-PTGS | No resistance | (Zanek et al., 2008) |
| **Sweet orange** | *Citrus sinensis* | *RUTACEAE* | CPsV | Citrus psorosis virus | Ophiovirus | - SS RNA | 54k, 48k and 24k genes | S-PTGS | delay of symptom | (Reyes et al., 2011b) |
| **Sweet orange** | *Citrus sinensis* | *RUTACEAE* | CPsV | Citrus psorosis virus | Ophiovirus | - SS RNA | 54k, 48k and 24k genes | Hp-PTGS | delay of symptom | (Reyes et al., 2011a) |
| **Pepper** | *Capsicum annuum* | *SOLANACEAE* | ToMV | Tomato mosaic virus | Tobamovirus | + SS RNA | CP gene | CPMR/S-PTGS | substantially retarded | (Shin et al., 2002) |
| **Pepper** | *Capsicum annuum* | *SOLANACEAE* | CMV-Kor | Cucumber mosaic virus | Cucumovirus | + SS RNA | CP gene | CPMR/S-PTGS | substantially retarded | (Shin et al., 2002) |
| **Tobacco** | *Nicotiana benthamiana* | *SOLANACEAE* | APLPV | American plum line pattern virus | Ilarvirus | + SS RNA | CP gene | Hp-PTGS | Complete resistance | (Liu et al., 2007) |
| **Tobacco** | *Nicotiana benthamiana* | *SOLANACEAE* | BaMV | Bamboo mosaic virus | Potexvirus | **+SS RNA** | satRNA | S-PTGS | **High resistance** | (Lin et al., 2013) |
| **Tobacco** | *Nicotiana benthamiana* | *SOLANACEAE* | CBSV | Cassava brown streak virus | Ipomovirus | ssRNA | CP | Hp-PTGS | **High resistance** | (Patil et al., 2011) |
| **Tobacco** | *Nicotiana benthamiana* | *SOLANACEAE* | CBSUV | Cassava brown streak Uganda virus | Ipomovirus | ssRNA | CP | Hp-PTGS | **High resistance** | (Patil et al., 2011) |
| **Tobacco** | *Nicotiana benthamiana* | *SOLANACEAE* | CLCuBuV | Cotton leaf curl Burewala virus | Geminivirus | SS DNA | Pre coat protein (V2) | AMIR | Good resistance | (Ali et al., 2013) |
| **Tobacco** | *Nicotiana benthamiana* | *SOLANACEAE* | CLCuKoV | Cotton leaf curl Khokhran virus | Geminivirus | SS DNA | Repsyn130 | S-PTGS | Medium resistance | (Yousaf et al., 2013) |
| **Tobacco** | *Nicotiana benthamiana* | *SOLANACEAE* | CLCuMuB | Cotton leaf curl Multan virus | Geminivirus | SS DNA | Repsyn130 | S-PTGS | Medium resistance | (Yousaf et al., 2013) |
| **Tobacco** | *Nicotiana benthamiana* | *SOLANACEAE* | CMV | Cucumber mosaic virus | Cucumovirus | +SS RNA | RNA-2 and CP gene | Hp-PTGS | Resistant plants | (Chen et al., 2004) |
| **Tobacco** | *Nicotiana benthamiana* | *SOLANACEAE* | CMV | Cucumber mosaic virus | Cucumovirus | + SS RNA | RNA 1 | S-PTGS | Moderate resistance | (Canto and Palukaitis, 1998) |
| **Tobacco** | *Nicotiana benthamiana* | *SOLANACEAE* | CMV | Cucumber mosaic virus | Cucumovirus | + SS RNA | 2b gene | S-PTGS/Hp-PTGS | High resistance | (Qu et al., 2007) |
| **Tobacco** | *Nicotiana benthamiana* | *SOLANACEAE* | CTV | Citrus tristeza virus | Closterovirus | + SS RNA | CP-P20 | Hp-PTGS | Complete resistance | (Roy et al., 2006) |
| **Tobacco** | *Nicotiana benthamiana* | *SOLANACEAE* | CTV | Citrus tristeza virus | Closterovirus | + SS RNA | P23+4'UTR | Hp-PTGS | Resistant | (Batuman et al., 2006) |
| **Tobacco** | *Nicotiana benthamiana* | *SOLANACEAE* | GLRaV-2 | Grapevine leafroll associated virus-2 | Closterovirus | + SS RNA | Nib gene | S-PTGS | Complete resistance | (Ling et al., 2008) |
| **Tobacco** | *Nicotiana benthamiana* | *SOLANACEAE* | GRSV | Groundnut ringspot virus | Tospovirus | - SS RNA | N gene | Hp-PTGS | High resistance | (Bucher et al., 2006) |
| **Tobacco** | *Nicotiana benthamiana* | *SOLANACEAE* | PDV | Prune dwarf virus | Ilavirus | + SS RNA | CP gene | Hp-PTGS | Complete resistance | (Liu et al., 2007) |
| **Tobacco** | *Nicotiana benthamiana* | *SOLANACEAE* | PMV | Peach mosaic virus | Trichovirus | + SS RNA | CP gene | Hp-PTGS | Complete resistance | (Liu et al., 2007) |
| **Tobacco** | *Nicotiana benthamiana* | *SOLANACEAE* | PNRSV | Prunus necrotic ringspot virus | Ilavirus | + SS RNA | CP gene | Hp-PTGS | Complete resistance | (Liu et al., 2007) |
| **Tobacco** | *Nicotiana benthamiana* | *SOLANACEAE* | PPV | Plum pox virus | Potyvirus | + SS RNA | CP gene | Hp-PTGS | Complete resistance | (Liu et al., 2007) |
| **Tobacco** | *Nicotiana benthamiana* | *SOLANACEAE* | PPV | Plum pox virus | Potyvirus | + SS RNA | Hc-pro | S-PTGS | Resistant plants | (Barajas et al., 2004) |
| **Tobacco** | *Nicotiana benthamiana* | *SOLANACEAE* | PPV | Plum pox virus | Potyvirus | + SS RNA | P1,P3,HcPro | Hp-PTGS | Complete resistance | (Di Nicola-Negri et al., 2005) |
| **Tobacco** | *Nicotiana benthamiana* | *SOLANACEAE* | PPV | Plum pox virus | Potyvirus | +SS RNA | P1,P4,HcPro | Hp-PTGS | Complete resistance | (Di Nicola-Negri et al., 2010) |
| **Tobacco** | *Nicotiana benthamiana* | *SOLANACEAE* | PPV | Plum pox virus | Potyvirus | +SS RNA | NIa-NIb-CP | S-PTGS | immune | (Guo et al., 1998) |
| **Tobacco** | *Nicotiana benthamiana* | *SOLANACEAE* | PPV | Plum pox virus | Potyvirus | +SS RNA | CI | S-PTGS | Complete symptomless | (Wittner et al., 1998) |
| **Tobacco** | *Nicotiana benthamiana* | *SOLANACEAE* | PVA | Potato virus A | Potyvirus | + SS RNA | 5^/^ untraslated + CP | S-PTGS | Good resistance | (Savenkov and Valkonen, 2001) |
| **Tobacco** | *Nicotiana benthamiana* | *SOLANACEAE* | PVY | Potato virus Y | Potyvirus | + SS RNA | Nib gene | Hp-PTGS | Complete resistance | (Xu et al., 2009) |
| **Tobacco** | *Nicotiana benthamiana* | *SOLANACEAE* | PVY | Potato virus Y | Potyvirus | + SS RNA | CI,NIa, (NIb), and CP | AMIR | High resistance | (Song et al., 2014) |
| **Tobacco** | *Nicotiana benthamiana* | *SOLANACEAE* | TCSV | Tomato chlorotic spot virus | Tospovirus | - SS RNA | N gene | Hp-PTGS | High resistance | (Bucher et al., 2006) |
| **Tobacco** | *Nicotiana benthamiana* | *SOLANACEAE* | TCV | Turnip crinkle virus | Carmovirus | + SS RNA | Full length TCV genome | CPMR and PTGS | Delay symptoms & recovery | (Vasudevan et al., 2008) |
| **Tobacco** | *Nicotiana benthamiana* | *SOLANACEAE* | TEV | Tobacco etch virus | Potyvirus | + SS RNA | CP gene | S-PTGS | High to low resistance | (Voloudakis et al., 2005) |
| **Tobacco** | *Nicotiana benthamiana* | *SOLANACEAE* | ToRSV | Tomato ringspot virus | Nepovirus | + SS RNA | CP gene | Hp-PTGS | Complete resistance | (Liu et al., 2007) |
| **Tobacco** | *Nicotiana benthamiana* | *SOLANACEAE* | TSV | Tobacco streak virus | Ilarvirus | +SS RNA | CP gene | Hp-PTGS | Complete resistance | (Pradeep et al., 2012) |
| **Tobacco** | *Nicotiana benthamiana* | *SOLANACEAE* | TSWV | Tomato spotted wilt virus | Tospovirus | - SS RNA | nucleocapsid protein (N) | S-PTGS | Low level of resistance | (Sonoda, 2003) |
| **Tobacco** | *Nicotiana benthamiana* | *SOLANACEAE* | TSWV | Tomato spotted wilt virus | Tospovirus | - SS RNA | NSs gene | S-PTGS | Low level of resistance | (Sonoda and Tsumuki, 2004) |
| **Tobacco** | *Nicotiana benthamiana* | *SOLANACEAE* | TSWV | Tomato spotted wilt virus | Tospovirus | - SS RNA | N gene | Hp-PTGS | High resistance | (Bucher et al., 2006) |
| **Tobacco** | *Nicotiana benthamiana* | *SOLANACEAE* | TSWV | Tomato spotted wilt virus | Tospovirus | - SS RNA | N, CP | S-PTGS | High resistance | (Jan et al., 2000) |
| **Tobacco** | *Nicotiana benthamiana* | *SOLANACEAE* | TuMV | Turnip mosaic virus | Potyvirus | + SS RNA | CP gene | S-PTGS | High resistance | (Jan et al., 1999) |
| **Tobacco** | *Nicotiana benthamiana* | *SOLANACEAE* | WSMoV | Watermelon silver mottle virus | Tospovirus | - SS RNA | N gene | Hp-PTGS | High resistance | (Bucher et al., 2006) |
| **Tobacco** | *Nicotiana benthamiana* | *SOLANACEAE* | WSMoV | Watermelon silver mottle virus | Tospovirus | - SS RNA | Rep gene | AMIR | Complete resistance | (Kung et al., 2012) |
| **Tobacco** | *Nicotiana tabacum* | *SOLANACEAE* | APMoV | Andean potato mottle virus | Comovirus | + SS RNA | CP(partial) | S-PTGS | Immune | (Vaslin et al., 2001) |
| **Tobacco** | *Nicotiana tabacum* | *SOLANACEAE* | CMV | Cucumber mosaic virus | Cucumovirus | + SS RNA | RNA 1 | S-PTGS | Complete resistance | (Canto and Palukaitis, 2001) |
| **Tobacco** | *Nicotiana tabacum* | *SOLANACEAE* | CMV | Cucumber mosaic virus | Cucumovirus | + SS RNA | CP gene | Hp-PTGS | Complete resistance | (Kalantidis et al., 2002) |
| **Tobacco** | *Nicotiana tabacum* | *SOLANACEAE* | CMV | Cucumber mosaic virus | Cucumovirus | + SS RNA | Rep gene | S-PTGS | Good resistance | (Anderson et al., 1992) |
| **Tobacco** | *Nicotiana tabacum* | *SOLANACEAE* | CMV | Cucumber mosaic virus | Cucumovirus | + SS RNA | 2b | AMIR | Effective resistance | (Qu et al., 2007) |
| **Tobacco** | *Nicotiana tabacum* | *SOLANACEAE* | GRSV | Groundnut ringspot virus | Tospovirus | - SS RNA | N gene | S-PTGS | High resistance | (Prins et al., 1995) |
| **Tobacco** | *Nicotiana tabacum* | *SOLANACEAE* | MYMV-Vig | Mungbean yellow mosaic virus-Vigna | Geminivirus | SS DNA | CP, Rep, MP, NSP | S-PTGS | Complete resistance | (Shivaprasad et al., 2006) |
| **Tobacco** | *Nicotiana tabacum* | *SOLANACEAE* | PVY | Potato virus Y | Potyvirus | + SS RNA | Pro gene | Hp-PTGS | Immune | (Waterhouse et al., 1998) |
| **Tobacco** | *Nicotiana tabacum* | *SOLANACEAE* | PVY | Potato virus Y | Potyvirus | + SS RNA | CP gene | S-PTGS | Complete resistance | (Han et al., 1999) |
| **Tobacco** | *Nicotiana tabacum* | *SOLANACEAE* | PVY | Potato virus Y | Potyvirus | + SS RNA | CP gene | Hp-PTGS | Complete resistance | (Jiang et al., 2011) |
| **Tobacco** | *Nicotiana tabacum* | *SOLANACEAE* | PVY | Potato virus Y | Potyvirus | + SS RNA | CP gene | S-PTGS | Complete resistance | (Masmoudi et al., 2002) |
| **Tobacco** | *Nicotiana tabacum* | *SOLANACEAE* | PVY | Potato virus Y | Potyvirus | + SS RNA | CP gene | CPMR/S-PTGS | Complete resistance | (Ghosh et al., 2002) |
| **Tobacco** | *Nicotiana tabacum* | *SOLANACEAE* | TCSV | Tomato chlorotic spot virus | Tospovirus | - SS RNA | N gene | S-PTGS | High resistance | (Prins et al., 1995) |
| **Tobacco** | *Nicotiana tabacum* | *SOLANACEAE* | TEV | Tobacco etch virus | Potyvirus | + SS RNA | CP gene | CPMR and PDR | Susceptible to immune | (Lindbo et al., 1993) |
| **Tobacco** | *Nicotiana tabacum* | *SOLANACEAE* | TMV | Tobacco mosaic virus | Tobamovirus | + SS RNA | Whole genome | S-PTGS | Resistance break after some time | (Jada et al., 2014) |
| **Tobacco** | *Nicotiana tabacum* | *SOLANACEAE* | ToLCNDV | Tomato leaf curl New Delhi virus | Geminivirus | SS DNA | AC2 and AC4 | tasiRNA | Good resistance | (Singh et al., 2015) |
| **Tobacco** | *Nicotiana tabacum* | *SOLANACEAE* | ToLCNDV | Tomato leaf curl New Delhi virus | Geminivirus | SS DNA | AV1 and AV2 | AMIR | Highly tolerant | (Vu et al., 2013) |
| **Tobacco** | *Nicotiana tabacum* | *SOLANACEAE* | TRV | Tobacco rattle virus | Tobravirus | +SS RNA | 57-kDa (Rep) | S-PTGS | High resistance | (Vassilakos et al., 2008) |
| **Tobacco** | *Nicotiana tabacum* | *SOLANACEAE* | TSWV | Tomato spotted wilt virus | Tospovirus | - SS RNA | N gene | S-PTGS | High resistance | (Gielen et al., 1991) |
| **Tobacco** | *Nicotiana tabacum* | *SOLANACEAE* | TSWV | Tomato spotted wilt virus | Tospovirus | - SS RNA | N gene | S-PTGS | High resistance | (Prins et al., 1995) |
| **Tobacco** | *Nicotiana tabacum* | *SOLANACEAE* | TSWV | Tomato spotted wilt virus | Tospovirus | - SS RNA | N gene, Nsm gene | S-PTGS/AS-PTGS | High resistance | (Prins et al., 1996) |
| **Tobacco** | *Nicotiana tabacum* | *SOLANACEAE* | TSWV | Tomato spotted wilt virus | Tospovirus | - SS RNA | NSm gene | S-PTGS/AS-PTGS | Moderate | (Prins et al., 1997) |
| **Tomato** | *Lycopersicum esculentum* | *SOLANACEAE* | CMV | Cucumber mosaic virus | Cucumovirus | + SS RNA | REP gene (truncated) | S-PTGS | Complete resistance | (Nunome et al., 2002) |
| **Tobacco** | *Lycopersicum esculentum* | *SOLANACEAE* | CMV | Cucumber mosaic virus | Cucumovirus | + SS RNA | 2a, 2b gene, 3^/^ UTR | AMIR | High resistance | (Zhang et al., 2011a) |
| **Tomato** | *Nicotiana benthamiana* | *SOLANACEAE* | PVA | Potato virus A | Potyvirus | SS DNA | P1 or VPg cistron | S-PTGS | Resistant to recovery | (Germundsson and Valkonen, 2006) |
| **Tomato** | *Lycopersicum esculentum* | *SOLANACEAE* | ToLCV | Tomato leaf curl virus | Geminivirus | SS DNA | REP gene | **AS-PTGS** | High resistance | (Praveen et al., 2005) |
| **Tomato** | *Lycopersicum esculentum* | *SOLANACEAE* | ToLCNDV | Tomato leaf curl New Delhi virus | Geminivirus | SS DNA | AV1 and AV2 | AMIR | Highly tolerant | (Vu et al., 2013) |
| **Tomato** | *Lycopersicum esculentum* | *SOLANACEAE* | TSWV | Tomato spotted wilt virus | Tospovirus | - SS RNA | N gene | S-PTGS | Complete resistance | (Nervo et al., 2003) |
| **Tomato** | *Lycopersicum esculentum* | *SOLANACEAE* | TYLCV | Tomato yellow leaf curl virus | Geminivirus | SS DNA | 3^/^ end of Rep gene | Hp-PTGS | Immunity | (Fuentes et al., 2006) |
| **Potato** | *Lycopersicum esculentum* | *SOLANACEAE* | TYLCV-Is | Tomato yellow leaf curl virus-Israel | Geminivirus | SS DNA | Rep gene | S-PTGS | Resistant to immune | (Antignus et al., 2004) |
| **Potato** | *Solanum tuberosum* | *SOLANACEAE* | PLRV | Potato leaf roll virus | Polerovirus | + SS RNA | Rep gene | **S-PTGS** | Less disease incidence | (Arif et al., 2009) |
| **Potato** | *Solanum tuberosum* | *SOLANACEAE* | PLRV | Potato leaf roll virus | Polerovirus | + SS RNA | CP | **Hp-PTGS** | Immune | (Arif et al., 2012) |
| **Potato** | *Solanum tuberosum* | *SOLANACEAE* | PLRV | Potato leaf roll virus | Polerovirus | + SS RNA | Replicase gene | **S-PTGS** | Recovery | (Ehrenfeld et al., 2004) |
| **Potato** | *Solanum tuberosum* | *SOLANACEAE* | PVX | Potato virus X | Potexvirus | + SS RNA | ORF2 | **Hp-PTGS** | Immune | (Arif et al., 2012) |
| **Potato** | *Solanum tuberosum* | *SOLANACEAE* | PVX | Potato virus X | Potexvirus | + SS RNA | CP | **CPMR/S-PTGS** | High resistance | (Doreste et al., 2002) |
| **Potato** | *Solanum tuberosum* | *SOLANACEAE* | PVY | Potato virus Y | Potyvirus | + SS RNA | Cp gene (untranslatable) | **S-PTGS** | Less disease incidence | (Arif et al., 2009) |
| **Potato** | *Solanum tuberosum* | *SOLANACEAE* | PVY | Potato virus Y | Potyvirus | + SS RNA | HcPro | **Hp-PTGS** | Immune | (Arif et al., 2012) |
| **Potato** | *Solanum tuberosum* | *SOLANACEAE* | PVY | Potato virus Y | Potyvirus | + SS RNA | 3^/^ part of CP gene | **Hp-PTGS** | High resistance | (Missiou et al., 2004) |
| **Potato** | *Solanum tuberosum* | *SOLANACEAE* | TRV | Tobacco rattle virus | Tobravirus | + SS RNA | 57kda | **S-PTGS** | Fewer infection symptoms | (Melander, 2006) |
| **Maize** | *Zea mays* | *POACEAE* | MDMV | Maize dwarf mosaic virus | Potyvirus | +SS RNA | P1 | hp-PTGS | **highly resistant** | (Zhang et al., 2010) |
| **Maize** | *Zea mays* | *POACEAE* | MDMV | Maize dwarf mosaic virus | Potyvirus | +SS RNA | CP | hp-PTGS | **highly resistant** | (Zhang et al., 2011c) |
| **Maize** | *Zea mays* | *POACEAE* | MDMV | Maize dwarf mosaic virus | Potyvirus | +SS RNA | P1 | hp-PTGS | **enhanced resistance** | (Zhang et al., 2013) |
| **Maize** | *Zea mays* | *POACEAE* | MSV | Maize streak virus | Mastrevirus | SS DNA | mutated Rep gene | **PMR/S-PTGS** | **higher survival rates** | (Shepherd et al., 2007) |
| **Maize** | *Zea mays* | *POACEAE* | RBSDV | Rice black-streaked dwarf virus | Fijivirus | dsRNA | Rnc70 | **RNaseIII** | **High resistance** | (Cao et al., 2013) |
| **Wheat** | *Triticum aestivum* | *POACEAE* | WSMV | [Wheat streak mosaic virus](http://apps.webofknowledge.com/full_record.do?product=WOS&search_mode=GeneralSearch&qid=1&SID=1CBhuxSe2N2y1YFdqtU&page=2&doc=18&cacheurlFromRightClick=no) | Tritimovirus | +SS RNA | Nib gene | **S-PTGS** | **Only mild symptoms** | (Sivamani et al., 2000) |
| **Wheat** | *Triticum aestivum* | *POACEAE* | WSMV | [Wheat streak mosaic virus](http://apps.webofknowledge.com/full_record.do?product=WOS&search_mode=GeneralSearch&qid=1&SID=1CBhuxSe2N2y1YFdqtU&page=2&doc=18&cacheurlFromRightClick=no) | Tritimovirus | +SS RNA | CP | **S-PTGS (CPMR)** | **Less symptoms, recovery** | (Sivamani et al., 2002) |
| **Wheat** | *Triticum aestivum* | *POACEAE* | WSMV | [Wheat streak mosaic virus](http://apps.webofknowledge.com/full_record.do?product=WOS&search_mode=GeneralSearch&qid=1&SID=1CBhuxSe2N2y1YFdqtU&page=2&doc=18&cacheurlFromRightClick=no) | Tritimovirus | +SS RNA | Nia | hp-PTGS | **complete resistance** | (Fahim et al., 2010) |
| **Wheat** | *Triticum aestivum* | *POACEAE* | WSMV | [Wheat streak mosaic virus](http://apps.webofknowledge.com/full_record.do?product=WOS&search_mode=GeneralSearch&qid=1&SID=1CBhuxSe2N2y1YFdqtU&page=2&doc=18&cacheurlFromRightClick=no) | Tritimovirus | +SS RNA | 5'UTR-P1-HcPro-P3 | **AMIR** | **immune** | (Fahim et al., 2012) |
| **Wheat** | *Triticum aestivum* | *POACEAE* | WSMV | [Wheat streak mosaic virus](http://apps.webofknowledge.com/full_record.do?product=WOS&search_mode=GeneralSearch&qid=1&SID=1CBhuxSe2N2y1YFdqtU&page=2&doc=18&cacheurlFromRightClick=no) | Tritimovirus | +SS RNA | partial CP | hp-PTGS | **resistant** | (Cruz et al., 2014) |
| **Wheat** | *Triticum aestivum* | *POACEAE* | WYMV | Wheat yellow mosaic virus | Bymovirus | +SS RNA | NIb replicase | AS-PTGS | broad-spectrum resistance | (Chen et al., 2014) |
| Rice | Oryza sativa | POACEAE | RBSDV | Rice black-streaked dwarf virus | Fijivirus | dsRNA | S1, S2, S6 and S10 | hp-PTGS | durable resistance | (Wang et al., 2016) |
| Rice | Oryza sativa | POACEAE | RBSDV | Rice black-streaked dwarf virus | Reovirus | dsRNA |  |  |  | (Sasaya et al., 2014) |
| Rice | Oryza sativa | POACEAE | RDV | Rice dwarf virus | Reovirus | dsRNA | Pns6, P8, Pns12 |  |  | (Sasaya et al., 2014) |
| Rice | Oryza Sativa | POACEAE | RDV | Rice dwarf virus | Phytoreovirus | dsRNA | Segment 8 of RDV | hp-PTGS | High resistance | (Ma et al., 2004) |
| Rice | Oryza sativa | POACEAE | RGDV | Rice gall dwarf virus | Reovirus | dsRNA |  |  |  | (Sasaya et al., 2014) |
| Rice | Oryza sativa | POACEAE | RGSV | Rice grassy stunt virus | Tenuivirus | -ssRNA |  |  |  | (Sasaya et al., 2014) |
| Rice | Oryza sativa | POACEAE | RHBV | Rice hoja blanca virus | Tenuivirus | -ssRNA |  |  |  | (Sasaya et al., 2014) |
| Rice | Oryza sativa | POACEAE | RSV | Rice stripe virus | Tenuivirus | -ssRNA | CP or SP | hp-PTGS | strongly resistant | (Jiang et al., 2013) |
| Rice | Oryza sativa | POACEAE | RSV | Rice stripe virus | Tenuivirus | -ssRNA |  |  |  | (Sasaya et al., 2014) |
| Rice | Oryza Sativa | POACEAE | RSV | Rice stripe virus | Tenuivirus | -ssRNA | CP and SP gene | hp-PTGS | High resistance | (Ma et al., 2011) |
| Rice | Oryza Sativa | POACEAE | RSV | Rice stripe virus | Tenuivirus | -ssRNA | pC2, pC3, pC4, p4 | hp-PTGS | Immune | (Shimizu et al., 2011) |
| Rice | Oryza sativa | POACEAE | RTBV | Rice tungro bacilliform virus | Tungrovirus | dsDNA RT | ORF IV | hp-PTGS | substantial improvements | (Valarmathi et al., 2016) |
| Rice | Oryza sativa | POACEAE | RTBV | Rice tungro bacilliform virus | Tungrovirus | dsDNA RT | ORF IV | hp-PTGS | substantial improvements | (Roy et al., 2012) |
| Rice | Oryza Sativa | POACEAE | RTBV | Rice tungro bacilliform virus | Tungro virus | dsDNA RT | CP gene | CPMR | Reduced viral titer | (Ganesan et al., 2009) |
| Rice | Oryza Sativa | POACEAE | RTBV | Rice tungro bacilliform virus | Tungro virus | dsDNA RT | ORF IV | hp-PTGS | Mild symptoms of disease | (Tyagi et al., 2008) |
| Rice | Oryza Sativa | POACEAE | RTSV | Rice tungro spherical virus | Waikavirus | +ss RNA | ORF IV | hp-PTGS | Substantial improvement | (Valarmathi et al., 2016) |
| Rice | Oryza Sativa | POACEAE | RTSV | Rice tungro spherical virus | Waikavirus | +ss RNA | ORF IV | hp-PTGS | Mild symptoms of disease | (Roy et al., 2012) |
| **Peanut** | *Arachis hypogaea* | *FABACEAE* | PStV | Peanut stripe virus | Potyvirus | +SS RNA | CP | S-PTGS | High resistance | (Higgins et al., 2004) |
| **Peanut** | *Arachis hypogaea* | *FABACEAE* | TSV | Tobacco streak virus | Ilarvirus | +SS RNA | CP gene | CPMR | Complete resistance | (Mehta et al., 2013) |
| **Peanut** | *Arachis hypogaea* | *FABACEAE* | PBNV | Peanut bud necrosis virus | Tospovirus | - SS RNA | N gene | PMR | Partial and non durable | (Rao et al., 2013), |
| **Peanut** | *Arachis hypogaea* | *FABACEAE* | TSWV | Tomaot spotted wilt virus | Tospovirus | - SS RNA | Nucleocapsid protein | PMR | Lower infection rate | (Yang et al., 2004) |
| **White clover** | *Trifolium repens* | *FABACEAE* | WCMV | White clover mosaic virus | Potexvirus | +SS RNA | Replicase gene | Hp-PTGS | High resistance | (Ludlow et al., 2009) |
| **Soybean** | *Glycine max* | *FABACEAE* | SMV | Soybean mosaic virus | Potyvirus | +SS RNA | Hc-pro gene | Hp-PTGS | Low to high | (Gao et al., 2015) |
| **Soybean** | *Glycine max* | *FABACEAE* | SMV | Soybean mosaic virus | Potyvirus | +SS RNA | CP gene and 3^/^ UTR | S-PTGS | Highly resistant | (Wang et al., 2001) |
| **Soybean** | *Glycine max* | *FABACEAE* | AMV | Alfalfa mosaic virus | Alfamovirus | +SS RNA | Rep | Hp-PTGS | strong systemic resistance to mixed infection | (Zhang et al., 2011b) |
| **Soybean** | *Glycine max* | *FABACEAE* | BPMV | Bean pod mottle virus | Comovirus | +SS RNA | Rep | Hp-PTGS |  | (Zhang et al., 2011b) |
| **Soybean** | *Glycine max* | *FABACEAE* | SMV | Soybean mosaic virus | Potyvirus | +SS RNA | Rep | Hp-PTGS |  | (Zhang et al., 2011b) |
| **common beans** | [*Phaseolus vulgaris*](https://en.wikipedia.org/wiki/Phaseolus) | *FABACEAE* | BGMV | Bean golden mosaic virus | Begomovirus | SS DNA | rep/AC1 | Hp-PTGS | completely resistant | (Aragao et al., 2013; Faria et al., 2014) |
| **common beans** | [*Phaseolus vulgaris*](https://en.wikipedia.org/wiki/Phaseolus) | *FABACEAE* | BGMV | Bean golden mosaic virus | Begomovirus | SS DNA | rep/AC1 | S-PTGS | partial resistant | (Faria et al., 2006) |
| **Sweet Potato** | *Ipomoea batatas* | *CONVOVULACEAE* | SPCSV | Sweet potato chlorotic stunt virus | Crinivirus | +SS RNA | RdRp gene | Hp-PTGS | Reduced symptoms | (Kreuze et al., 2008) |
| **Cantaloupe** | *Cucumis melo* | *CUCURBITACEAE* | PRSV-W | Papaya ringspot virus type W | Potyvirus | +SS RNA | CP gene | Hp-PTGS | High resistance | (Krubphachaya et al., 2007) |
| Oriental melon | *Cucumis melo* | *CUCURBITACEAE* | PRSV-W | Papaya ringspot virus | Potyvirus | +SS RNA | CP | S-PTGS | complete immunity | (Wu et al., 2010) |
| Oriental melon | *Cucumis melo* | *CUCURBITACEAE* | ZYMV | Zucchini yellow mosaic virus | Potyvirus | +SS RNA | CP | S-PTGS | complete immunity | (Wu et al., 2010) |
| **Cucumber** | *Cucumis sativus* | *CUCURBITACEAE* | CFMMV | Cucumber fruit mottle mosaic virus | Tobamovirus | +SS RNA | 54-kDa | S-PTGS | immune | (Gal-On et al., 2005) |
| **zucchini squash** | *Cucurbita pepo* | *CUCURBITACEAE* | SqMV | Squash mosaic virus | Cocomvirus | +SS RNA | CP | S-PTGS | Highly resistant | (Pang et al., 2000) |
| Watermelon | *Citrullus lanatus* | *CUCURBITACEAE* | WSMoV | Watermelon silver mottle virus | Tospovirus | - SS RNA | N | S-PTGS | resistant to single and mix infection | (Lin et al., 2012) |
| Watermelon | *Citrullus lanatus* | *CUCURBITACEAE* | CMV | Cucumber mosaic virus | Cucumovirus | +SS RNA | CP | S-PTGS |  | (Lin et al., 2012) |
| Watermelon | *Citrullus lanatus* | *CUCURBITACEAE* | CGMMV | Cucumber green mottle mosaic virus | Tobamovirus | +SS RNA | CP | S-PTGS |  | (Lin et al., 2012) |
| Watermelon | *Citrullus lanatus* | *CUCURBITACEAE* | WMV | Watermelon mosaic virus | Potyvirus | +SS RNA | CP | S-PTGS |  | (Lin et al., 2012) |
| **Sunflower** | *Helianthus annuus,* | *ASTERACEAE* | TSV | Tobacco streak virus | Ilarvirus | +SS RNA | CP gene | Hp-PTGS | Complete resistance | (Pradeep et al., 2012) |
| **Chrysanthemum** | *Dendranthema grandiflora* | *ASTERACEAE* | TSWV | Tomato spotted wilt virus | Tospovirus | -SS RNA | N gene | S-PTGS | high levels of resistance | (Sherman et al., 1998) |
| **Plum** | *Prunus mariana* | *ROSACEAE* | PPV | Plum Pox virus | Potyvirus | +SS RNA | CP gene | S-PTGS | highly resistant | (Scorza et al., 2001) |
| **Plum** | *Prunus domestica* | *ROSACEAE* | PPV | Plum Pox virus | Potyvirus | +SS RNA | CP gene | hp-PTGS | resistant | (Ravelonandro et al., 2014) |
| **Plum** | *Prunus domestica* | *ROSACEAE* | PPV | Plum Pox virus | Potyvirus | +SS RNA | CP gene | hp-PTGS | resistant | (Hily et al., 2007) |
| **Orchid** | *Dendrobium Sonia* | *ORCHIDACEAE* | CymMV | *Cymbidium Mosaic Virus* | Tombusvirus | +SS RNA | CP | S-PTGS | Recovery | (Petchthai et al., 2015) |
| **Orchid** | *Dendrobium* | *ORCHIDACEAE* | CymMV | *Cymbidium Mosaic Virus* | Tombusvirus | +SS RNA | CP | S-PTGS | much milder symptoms | (Chang et al., 2005) |
| **Orchid** | *Phalaenopsis orchid* | *ORCHIDACEAE* | CymMV | *Cymbidium Mosaic Virus* | Tombusvirus | +SS RNA | CP | S-PTGS | reistant | (Liao et al., 2004) |
| **Orchid** | *Phalaenopsis orchid* | *ORCHIDACEAE* | CymMV | *Cymbidium Mosaic Virus* | Tombusvirus | +SS RNA | CP | S-PTGS | reistant | (Chan et al., 2005) |
| **Cassava** | *Manihot esculenta* | *EUPHORBIACEAE* | *ACMV* | *African cassava mosaic virus* | *Begmovirus* | *SS DNA* | *AC1 gene* | *S-PTGS* | High resistance | (Chellappan et al., 2004) |
| **Cassava** | *Manihot esculenta* | *EUPHORBIACEAE* | *SLCMV* | *Sri Lankan cassava mosaic virus* | *Begmovirus* | *SS DNA* | *between the AV2 and AV1* | hp-PTGS | high levels of resistance | *(Ntui et al., 2015)* |
| **Cassava** | *Manihot esculenta* | *EUPHORBIACEAE* | *ACMV* | *African cassava mosaic virus* | *Begmovirus* | *SS DNA* | *bidrectional promoter* | hp-TGS | dramatically attenuated | *(Vanderschuren et al., 2007)* |
| **Cassava** | *Manihot esculenta* | *EUPHORBIACEAE* | *CBSUV* | *Cassava brown streak Uganda virus* | *Ipomovirus* | *+SSRNA* | *CP* | Hp-PTGS | Complete resistance | *(Yadav et al., 2011)* |
| **Papaya** | *Carica papaya* | *CARICACEAE* | PRSV | *Papaya ringspot virus* | Potyvirus | +SSRNA | CP | S-PTGS | Complete resistance | (Fitch et al., 1992) |
| **Papaya** | *Carica papaya* | *CARICACEAE* | PRSV | *Papaya ringspot virus* | Potyvirus | +SSRNA | Rep partial | S-PTGS | resistant | (Chen et al., 2001) |
| **Papaya** | *Carica papaya* | *CARICACEAE* | PRSV | *Papaya ringspot virus* | Potyvirus | +SSRNA | CP | S-PTGS | immune | (Lines et al., 2002) |
| **Papaya** | *Carica papaya* | *CARICACEAE* | PRSV | *Papaya ringspot virus* | Potyvirus | +SSRNA | CP | S-PTGS | High resistance to immune | (Bau et al., 2003) |
| **Papaya** | *Carica papaya* | *CARICACEAE* | PRSV | *Papaya ringspot virus* | Potyvirus | +SSRNA | CP | S-PTGS | highly resistant | (Davis and Ying, 2004) |
| **Papaya** | *Carica papaya* | *CARICACEAE* | PRSV | *Papaya ringspot virus* | Potyvirus | +SSRNA | CP | S-PTGS | Complete high resistance | (Tennant et al., 2005) |
| **Papaya** | *Carica papaya* | *CARICACEAE* | PRSV | *Papaya ringspot virus* | Potyvirus | +SSRNA | CP | S-PTGS | high levels of resistance to heterologous PRSV strains | (Kung et al., 2009) |
| **Papaya** | *Carica papaya* | *CARICACEAE* | PLDMV | *Papaya leaf-distortion mosaic virus* | Potyvirus | +SSRNA | CP | S-PTGS |  | (Kung et al., 2009) |
| **Papaya** | *Carica papaya* | *CARICACEAE* | PRSV | *Papaya ringspot virus* | Potyvirus | +SSRNA | CP | CPMR | No resistance | (Roberts et al., 2014) |
| **Papaya** | *Carica papaya* | *CARICACEAE* | PRSV | *Papaya ringspot virus* | Potyvirus | +SSRNA | HCPro | S-PTGS | resistant to more virulent strain | (Kung et al., 2015) |
| **Banana** | *Musa  paradisiaca* | *MUSACEAE* | BBTV | *Banana bunchy top virus* | Babuvirus | SS DNA | Rep gene | hp-PTGS | Complete resistance | (Elayabalan et al., 2013) |
| **Banana** | *Musa  paradisiaca* | *MUSACEAE* | BBTV | *Banana bunchy top virus* | Babuvirus | SS DNA | Rep gene | hp-PTGS | Complete resistance | (Shekhawat et al., 2012) |

# Supplemental References:

Ali, I., Amin, I., Briddon, R.W., Mansoor, S., 2013. Artificial microRNA-mediated resistance against the monopartite begomovirus Cotton leaf curl Burewala virus. Virol J 10, 231.

Anderson, J.M., Palukaitis, P., Zaitlin, M., 1992. A defective replicase gene induces resistance to cucumber mosaic virus in transgenic tobacco plants. Proc Natl Acad Sci U S A 89, 8759-8763.

Antignus, Y., Vunsh, R., Lachman, O., Pearlsman, M., Maslenin, L., Hananya, U., Rosner, A., 2004. Truncated Rep gene originated from Tomato yellow leaf curl virus-Israel [Mild] confers strain-specific resistance in transgenic tomato. Ann Appl Biol 144, 39-44.

Aragao, F.J.L., Nogueira, E.O.P.L., Tinoco, M.L.P., Faria, J.C., 2013. Molecular characterization of the first commercial transgenic common bean immune to the Bean golden mosaic virus. J Biotechnol 166, 42-50.

Arif, M., Azhar, U., Arshad, M., Zafar, Y., Mansoor, S., Asad, S., 2012. Engineering broad-spectrum resistance against RNA viruses in potato. Transgenic Res 21, 303-311.

Arif, M., Thomas, P.E., Crosslin, J.M., Brown, C.R., 2009. Development of Molecular Resistance in Potato against Potato Leaf Roll Virus and Potato Virus Y through Agrobacterium-Mediated Double Transgenesis. Pak J Bot 41, 945-954.

Barajas, D., Tenllado, F., Gonzalez-Jara, P., Martinez-Garcia, B., Atencio, F.A., Diaz-Ruiz, J.R., 2004. Resistance to Plum pox virus (PPV) in Nicotiana benthamlana plants transformed with the PPVHC-PRO silencing suppressor gene. J Plant Pathol 86, 239-248.

Batuman, O., Mawassi, M., Bar-Joseph, M., 2006. Transgenes consisting of a dsRNA of an RNAi suppressor plus the 3 ' UTR provide resistance to Citrus tristeza virus sequences in Nicotiana benthamiana but not in citrus. Virus Genes 33, 319-327.

Bau, H.J., Cheng, Y.I.H., Yu, T.A., Yang, J.S., Yeh, S.D., 2003. Broad-spectrum resistance to different geographic strains of Papaya ringspot virus in coat protein gene transgenic papaya. Phytopathology 93, 112-120.

Bucher, E., Lohuis, D., van Poppel, P.M., Geerts-Dimitriadou, C., Goldbach, R., Prins, M., 2006. Multiple virus resistance at a high frequency using a single transgene construct. J Gen Virol 87, 3697-3701.

Canto, T., Palukaitis, P., 1998. Transgenically expressed cucumber mosaic virus RNA 1 simultaneously complements replication of cucumber mosaic virus RNAs 2 and 3 and confers resistance to systemic infection. Virology 250, 325-336.

Canto, T., Palukaitis, P., 2001. A cucumber mosaic virus (CMV) RNA 1 transgene mediates suppression of the homologous viral RNA 1 constitutively and prevents CMV entry into the phloem. J Virol 75, 9114-9120.

Cao, X.L., Lu, Y.G., Di, D.P., Zhang, Z.Y., Liu, H., Tian, L.Z., Zhang, A.H., Zhang, Y.J., Shi, L.D., Guo, B.H., Xu, J., Duan, X.F., Wang, X.B., Han, C.G., Miao, H.Q., Yu, J.L., Li, D.W., 2013. Enhanced Virus Resistance in Transgenic Maize Expressing a dsRNA-Specific Endoribonuclease Gene from E. coli. Plos One 8.

Chan, Y.L., Lin, K.H., Sanjaya, Liao, L.J., Chen, W.H., Chan, M.T., 2005. Gene stacking in Phalaenopsis orchid enhances dual tolerance to pathogen attack. Transgenic Research 14, 279-288.

Chang, C., Chen, Y.C., Hsu, Y.H., Wu, J.T., Hu, C.C., Chang, W.C., Lin, N.S., 2005. Transgenic resistance to Cymbidium mosaic virus in Dendrobium expressing the viral capsid protein gene. Transgenic Research 14, 41-46.

Chellappan, P., Masona, M.V., Vanitharani, R., Taylor, N.J., Fauquet, C.M., 2004. Broad spectrum resistance to ssDNA viruses associated with transgene-induced gene silencing in cassava. Plant Molecular Biology 56, 601-611.

Chen, G., Ye, C.M., Huang, J.C., Yu, M., Li, B.J., 2001. Cloning of the papaya ringspot virus (PRSV) replicase gene and generation of PRSV-resistant papayas through the introduction of the PRSV replicase gene. Plant Cell Rep 20, 272-277.

Chen, M., Sun, L., Wu, H., Chen, J., Ma, Y., Zhang, X., Du, L., Cheng, S., Zhang, B., Ye, X., Pang, J., Zhang, X., Li, L., Andika, I.B., Chen, J., Xu, H., 2014. Durable field resistance to wheat yellow mosaic virus in transgenic wheat containing the antisense virus polymerase gene. Plant Biotechnol J 12, 447-456.

Chen, Y.K., Lohuis, D., Goldbach, R., Prins, M., 2004. High frequency induction of RNA-mediated resistance against Cucumber mosaic virus using inverted repeat constructs. Molecular Breeding 14, 215-226.

Cruz, L.F., Rupp, J.L.S., Trick, H.N., Fellers, J.P., 2014. Stable resistance to Wheat streak mosaic virus in wheat mediated by RNAi. In Vitro Cell Dev-Pl 50, 665-672.

Davis, M.J., Ying, Z.T., 2004. Development of papaya breeding lines with transgenic resistance to Papaya ringspot virus. Plant Disease 88, 352-358.

Di Nicola-Negri, E., Brunetti, A., Tavazza, M., Ilardi, V., 2005. Hairpin RNA-mediated silencing of Plum pox virus P1 and HC-Pro genes for efficient and predictable resistance to the virus. Transgenic Research 14, 989-994.

Di Nicola-Negri, E., Tavazza, M., Salandri, L., Ilardi, V., 2010. Silencing of Plum pox virus 5'UTR/P1 sequence confers resistance to a wide range of PPV strains. Plant Cell Rep 29, 1435-1444.

Dominguez, A., Fagoaga, C., Navarro, L., Moreno, P., Pena, L., 2002. Regeneration of transgenic citrus plants under non selective conditions results in high-frequency recovery of plants with silenced transgenes. Mol Genet Genomics 267, 544-556.

Doreste, V., Ramos, P.L., Enriquez, G.A., Rodriguez, R., Peral, R., Pujol, M., 2002. Transgenic potato plants expressing the potato virus X (PVX) coat protein gene developed resistance to the viral infection. Phytoparasitica 30, 177-185.

Ehrenfeld, N., Romano, E., Serrano, C., Arce-Johnson, P., 2004. Replicase mediated resistance against Potato Leafroll Virus in potato Desiree plants. Biol Res 37, 71-82.

Elayabalan, S., Kalaiponmani, K., Subramaniam, S., Selvarajan, R., Panchanathan, R., Muthuvelayoutham, R., Kumar, K.K., Balasubramanian, P., 2013. Development of Agrobacterium-mediated transformation of highly valued hill banana cultivar Virupakshi (AAB) for resistance to BBTV disease. World J Microbiol Biotechnol 29, 589-596.

Fahim, M., Ayala-Navarrete, L., Millar, A.A., Larkin, P.J., 2010. Hairpin RNA derived from viral NIa gene confers immunity to wheat streak mosaic virus infection in transgenic wheat plants. Plant Biotechnol J 8, 821-834.

Fahim, M., Millar, A.A., Wood, C.C., Larkin, P.J., 2012. Resistance to Wheat streak mosaic virus generated by expression of an artificial polycistronic microRNA in wheat. Plant Biotechnol J 10, 150-163.

Faria, J.C., Albino, M.M.C., Dias, B.B.A., Cancado, L.J., da Cunha, N.B., Silva, L.D., Vianna, G.R., Aragao, F.J.L., 2006. Partial resistance to Bean golden mosaic virus in a transgenic common bean (Phaseolus vulgar L.) line expressing a mutated rep gene. Plant Sci 171, 565-571.

Faria, J.C., Valdisser, P.A.M.R., Nogueira, E.O.P.L., Aragao, F.J.L., 2014. RNAi-based Bean golden mosaic virus-resistant common bean (Embrapa 5.1) shows simple inheritance for both transgene and disease resistance. Plant Breeding 133, 649-653.

Febres, V.J., Lee, R.F., Moore, G.A., 2008. Transgenic resistance to Citrus tristeza virus in grapefruit. Plant Cell Rep 27, 93-104.

Fitch, M.M.M., Manshardt, R.M., Gonsalves, D., Slightom, J.L., Sanford, J.C., 1992. Virus Resistant Papaya Plants Derived from Tissues Bombarded with the Coat Protein Gene of Papaya Ringspot Virus. Nature Biotechnology 10, 1466-1472.

Fuentes, A., Ramos, P.L., Fiallo, E., Callard, D., Sanchez, Y., Peral, R., Rodriguez, R., Pujol, M., 2006. Intron-hairpin RNA derived from replication associated protein C1 gene confers immunity to tomato yellow leaf curl virus infection in transgenic tomato plants. Transgenic Res 15, 291-304.

Gal-On, A., Wolf, D., Antignus, Y., Patlis, L., Ryu, K.H., Min, B.E., Pearlsman, M., Lachman, O., Gaba, V., Wang, Y., Shiboleth, Y.M., Yang, J., Zelcer, A., 2005. Transgenic cucumbers harboring the 54-kDa putative gene of Cucumber fruit mottle mosaic tobamovirus are highly resistant to viral infection and protect non-transgenic scions from soil infection. Transgenic Res 14, 81-93.

Ganesan, U., Suri, S.S., Rajasubramaniam, S., Rajam, M.V., Dasgupta, I., 2009. Transgenic expression of coat protein gene of Rice tungro bacilliform virus in rice reduces the accumulation of viral DNA in inoculated plants. Virus Genes 39, 113-119.

Gao, L., Ding, X., Li, K., Liao, W., Zhong, Y., Ren, R., Liu, Z., Adhimoolam, K., Zhi, H., 2015. Characterization of Soybean mosaic virus resistance derived from inverted repeat-SMV-HC-Pro genes in multiple soybean cultivars. Theor Appl Genet 128, 1489-1505.

Germundsson, A., Valkonen, J.P., 2006. P1- and VPg-transgenic plants show similar resistance to Potato virus A and may compromise long distance movement of the virus in plant sections expressing RNA silencing-based resistance. Virus Res 116, 208-213.

Ghosh, S.B., Nagi, L.H.S., Ganapathi, T.R., Khurana, S.M.P., Bapat, V.A., 2002. Cloning and sequencing of potato virus Y coat protein gene from an Indian isolate and development of transgenic tobacco for PVY resistance. Curr Sci India 82, 855-859.

Gielen, J.J., de Haan, P., Kool, A.J., Peters, D., Van Grinsven, M.Q., Goldbach, R.W., 1991. Engineered Resistance to Tomato Spotted Wilt Virus, a Negative–Strand RNA Virus. Nature Biotechnology 9, 1363-1367.

Guo, H.S., Cervera, M.T., Garcia, J.A., 1998. Plum pox potyvirus resistance associated to transgene silencing that can be stabilized after different number of plant generations. Gene 206, 263-272.

Han, S.J., Cho, H.S., You, J.S., Nam, Y.W., Park, E.K., Shin, J.S., Park, Y.I., Park, W.M., Paek, K.H., 1999. Gene silencing-mediated resistance in transgenic tobacco plants carrying potato virus Y coat protein gene. Mol Cells 9, 376-383.

Higgins, C.M., Hall, R.M., Mitter, N., Cruickshank, A., Dietzgen, R.G., 2004. Peanut stripe potyvirus resistance in peanut (Arachis hypogaea L.) plants carrying viral coat protein gene sequences. Transgenic Res 13, 59-67.

Hily, J.M., Ravelonandro, M., Damsteegt, V., Bassett, C., Petri, C., Liu, Z., Scorza, R., 2007. Plum pox virus coat protein gene Intron-hairpin-RNA (ihpRNA) constructs provide resistance to plum pox virus in Nicotiana benthamiana and Prunus domestica. J Am Soc Hortic Sci 132, 850-858.

Iwanami, T., Shimizu, T., Ito, T., Hirabayashi, T., 2004. Tolerance to Citrus mosaic virus in transgenic trifoliate orange lines harboring capsid polyprotein gene. Plant Disease 88, 865-868.

Jada, B., Soitamo, A.J., Siddiqui, S.A., Murukesan, G., Aro, E.M., Salakoski, T., Lehto, K., 2014. Multiple Different Defense Mechanisms Are Activated in the Young Transgenic Tobacco Plants Which Express the Full Length Genome of the Tobacco Mosaic Virus, and Are Resistant against this Virus. Plos One 9.

Jan, F.J., Fagoaga, C., Pang, S.Z., Gonsalves, D., 2000. A single chimeric transgene derived from two distinct viruses confers multi-virus resistance in transgenic plants through homology-dependent gene silencing. J Gen Virol 81, 2103-2109.

Jan, F.J., Pang, S.Z., Fagoaga, C., Gonsalves, D., 1999. Turnip mosaic potyvirus resistance in Nicotiana benthamiana derived by post-transcriptional gene silencing. Transgenic Res 8, 203-213.

Jiang, F., Wu, B., Zhang, C., Song, Y., An, H., Zhu, C., Wen, F., 2011. Special origin of stem sequence influence the resistance of hairpin expressing plants against PVY. Biol Plantarum 55, 528-535.

Jiang, Y., Sun, L., Jiang, M., Li, K., Song, Y., Zhu, C., 2013. Production of marker-free and RSV-resistant transgenic rice using a twin T-DNA system and RNAi. J Biosci 38, 573-581.

Kalantidis, K., Psaradakis, S., Tabler, M., Tsagris, M., 2002. The occurrence of CMV-specific short Rnas in transgenic tobacco expressing virus-derived double-stranded RNA is indicative of resistance to the virus. Mol Plant Microbe Interact 15, 826-833.

Kreuze, J.F., Klein, I.S., Lazaro, M.U., Chuquiyuri, W.J.C., Morgan, G.L., Mejia, P.G.C., Ghislain, M., Valkonen, J.P.T., 2008. RNA silencing-mediated resistance to a crinivirus (Closteroviridae) in cultivated sweetpotato (Ipomoea batatas L.) and development of sweetpotato virus disease following co-infection with a potyvirus. Molecular Plant Pathology 9, 589-598.

Krubphachaya, P., Juricek, M., Kertbundit, S., 2007. Induction of RNA-mediated resistance to papaya ringspot virus type W. J Biochem Mol Biol 40, 404-411.

Kung, Y.J., Bau, H.J., Wu, Y.L., Huang, C.H., Chen, T.M., Yeh, S.D., 2009. Generation of Transgenic Papaya with Double Resistance to Papaya ringspot virus and Papaya leaf-distortion mosaic virus. Phytopathology 99, 1312-1320.

Kung, Y.J., Lin, S.S., Huang, Y.L., Chen, T.C., Harish, S.S., Chua, N.H., Yeh, S.D., 2012. Multiple artificial microRNAs targeting conserved motifs of the replicase gene confer robust transgenic resistance to negative-sense single-stranded RNA plant virus. Mol Plant Pathol 13, 303-317.

Kung, Y.J., You, B.J., Raja, J.A.J., Chen, K.C., Huang, C.H., Bau, H.J., Yang, C.F., Huang, C.H., Chang, C.P., Yeh, S.D., 2015. Nucleotide Sequence-Homology-Independent Breakdown of Transgenic Resistance by More Virulent Virus Strains and a Potential Solution. Sci Rep-Uk 5.

Liao, L.J., Pan, I.C., Chan, Y.L., Hsu, Y.H., Chen, W.H., Chan, M.T., 2004. Transgene silencing in Phalaenopsis expressing the coat protein of Cymbidium Mosaic Virus is a manifestation of RNA-mediated resistance. Molecular Breeding 13, 229-242.

Lin, C.Y., Ku, H.M., Chiang, Y.H., Ho, H.Y., Yu, T.A., Jan, F.J., 2012. Development of transgenic watermelon resistant to Cucumber mosaic virus and Watermelon mosaic virus by using a single chimeric transgene construct. Transgenic Research 21, 983-993.

Lin, K.Y., Hsu, Y.H., Chen, H.C., Lin, N.S., 2013. Transgenic resistance to Bamboo mosaic virus by expression of interfering satellite RNA. Molecular Plant Pathology 14, 693-707.

Lindbo, J.A., Silva-Rosales, L., Proebsting, W.M., Dougherty, W.G., 1993. Induction of a Highly Specific Antiviral State in Transgenic Plants: Implications for Regulation of Gene Expression and Virus Resistance. Plant Cell 5, 1749-1759.

Lines, R.E., Persley, D., Dale, J.L., Drew, R., Bateson, M.F., 2002. Genetically engineered immunity to Papaya ringspot virus in Australian papaya cultivars. Molecular Breeding 10, 119-129.

Ling, K.S., Zhu, H.Y., Gonsalves, D., 2008. Resistance to Grapevine leafroll associated virus-2 is conferred by post-transcriptional gene silencing in transgenic Nicotiana benthamiana. Transgenic Res 17, 733-740.

Liu, Z.R., Scorza, R., Hily, J.M., Scott, S.W., James, D., 2007. Engineering resistance to multiple Prunus fruit viruses through expression of chimeric hairpins. J Am Soc Hortic Sci 132, 407-414.

Loeza-Kuk, E., Gutierrez-Espinosa, M.A., Ochoa-Martinez, D.L., Villegas-Monter, A., Mora-Aguilera, G., Palacios-Torres, E.C., Perez-Molphe-Balch, E., 2011. RESISTANCE ANALYSIS IN GRAPEFRUIT AND MEXICAN LIME TRANSFORMED WITH the p25 Citrus tristeza virus GEN. Agrociencia-Mexico 45, 55-65.

Ludlow, E.J., Mouradov, A., Spangenberg, G.C., 2009. Post-transcriptional gene silencing as an efficient tool for engineering resistance to white clover mosaic virus in white clover (Trifolium repens). J Plant Physiol 166, 1557-1567.

Ma, J., Song, Y., Wu, B., Jiang, M., Li, K., Zhu, C., Wen, F., 2011. Production of transgenic rice new germplasm with strong resistance against two isolations of Rice stripe virus by RNA interference. Transgenic Res 20, 1367-1377.

Ma, Z.L., Yang, H.Y., Wang, R., Tien, P., 2004. Construct hairpin RNA to fight against rice dwarf virus. Acta Bot Sin 46, 332-336.

Masmoudi, K., Yacoubi, I., Hassairi, A., Elarbi, L.N., Ellouz, R., 2002. Tobacco plants transformed with an untranslatable form of the coat protein gene of the Potato virus Y are resistant to viral infection. Eur J Plant Pathol 108, 285-292.

Mehta, R., Radhakrishnan, T., Kumar, A., Yadav, R., Dobaria, J.R., Thirumalaisamy, P.P., Jain, R.K., Chigurupati, P., 2013. Coat protein-mediated transgenic resistance of peanut (Arachis hypogaea L.) to peanut stem necrosis disease through Agrobacterium-mediated genetic transformation. Indian J Virol 24, 205-213.

Melander, M., 2006. Potato transformed with a 57-kDa readthrough portion of the Tobacco rattle virus replicase gene displays reduced tuber symptoms when challenged by viruliferous nematodes. Euphytica 150, 123-130.

Missiou, A., Kalantidis, K., Boutla, A., Tzortzakaki, S., Tabler, M., Tsagris, M., 2004. Generation of transgenic potato plants highly resistant to potato virus Y (PVY) through RNA silencing. Molecular Breeding 14, 185-197.

Nervo, G., Cirillo, C., Accotto, G.P., Vaira, A.M., 2003. Characterisation of two tomato lines highly resistant to tomato spotted wilt virus following transformation with the viral nucleoprotein gene. J Plant Pathol 85, 139-144.

Ntui, V.O., Kong, K., Khan, R.S., Igawa, T., Janavi, G.J., Rabindran, R., Nakamura, I., Mii, M., 2015. Resistance to Sri Lankan Cassava Mosaic Virus (SLCMV) in Genetically Engineered Cassava cv. KU50 through RNA Silencing. Plos One 10.

Nunome, T., Fukumoto, F., Terami, F., Hanada, K., Hirai, M., 2002. Development of breeding materials of transgenic tomato plants with a truncated replicase gene of cucumber mosaic virus for resistance to the virus. Breeding Sci 52, 219-223.

Olivares-Fuster, O., Fleming, G.H., Albiach-Marti, M.R., Gowda, S., Dawson, W.O., Grosser, J.W., 2003. Citrus tristeza virus (CTV) resistance in transgenic citrus based on virus challenge of protoplasts. In Vitro Cell Dev-Pl 39, 567-572.

Pang, S.Z., Jan, F.J., Tricoli, D.M., Russell, P.F., Carney, K.J., Hu, J.S., Fuchs, M., Quemada, H.D., Gonsalves, D., 2000. Resistance to squash mosaic comovirus in transgenic squash plants expressing its coat protein genes. Molecular Breeding 6, 87-93.

Patil, B.L., Ogwok, E., Wagaba, H., Mohammed, I.U., Yadav, J.S., Bagewadi, B., Taylor, N.J., Kreuze, J.F., Maruthi, M.N., Alicai, T., Fauquet, C.M., 2011. RNAi-mediated resistance to diverse isolates belonging to two virus species involved in Cassava brown streak disease. Molecular Plant Pathology 12, 31-41.

Petchthai, U., Chuphrom, A., Huehne, P.S., 2015. Recovery of virus-infected Dendrobium orchids by constitutive expression of the cymbidium mosaic virus coat protein gene. Plant Cell Tiss Org 120, 597-606.

Pradeep, K., Satya, V.K., Selvapriya, M., Vijayasamundeeswari, A., Ladhalakshmi, D., Paranidharan, V., Rabindran, R., Samiyappan, R., Balasubramanian, P., Velazhahan, R., 2012. Engineering resistance against Tobacco streak virus (TSV) in sunflower and tobacco using RNA interference. Biol Plantarum 56, 735-741.

Praveen, S., Kushwaha, C.M., Mishra, A.K., Singh, V., Jain, R.K., Varma, A., 2005. Engineering tomato for resistance to tomato leaf curl disease using viral rep gene sequences. Plant Cell Tiss Org 83, 311-318.

Prins, M., de Haan, P., Luyten, R., van Veller, M., van Grinsven, M.Q., Goldbach, R., 1995. Broad resistance to tospoviruses in transgenic tobacco plants expressing three tospoviral nucleoprotein gene sequences. Mol Plant Microbe Interact 8, 85-91.

Prins, M., Kikkert, M., Ismayadi, C., de Graauw, W., de Haan, P., Goldbach, R., 1997. Characterization of RNA-mediated resistance to tomato spotted wilt virus in transgenic tobacco plants expressing NS(M) gene sequences. Plant Mol Biol 33, 235-243.

Prins, M., Resende Rde, O., Anker, C., van Schepen, A., de Haan, P., Goldbach, R., 1996. Engineered RNA-mediated resistance to tomato spotted wilt virus is sequence specific. Mol Plant Microbe Interact 9, 416-418.

Qu, J., Ye, J., Fang, R., 2007. Artificial microRNA-mediated virus resistance in plants. J Virol 81, 6690-6699.

Rao, S.C., Bhatnagar-Mathur, P., Kumar, P.L., Reddy, A.S., Sharma, K.K., 2013. Pathogen-derived resistance using a viral nucleocapsid gene confers only partial non-durable protection in peanut against peanut bud necrosis virus. Archives of Virology 158, 133-143.

Ravelonandro, M., Scorza, R., Michel, H.J., Briard, P., 2014. The efficiency of RNA interference for conferring stable resistance to plum pox virus. Plant Cell Tiss Org 118, 347-356.

Reyes, C.A., De Francesco, A., Pena, E.J., Costa, N., Plata, M.I., Sendin, L., Castagnaro, A.P., Garcia, M.L., 2011a. Resistance to Citrus psorosis virus in transgenic sweet orange plants is triggered by coat protein-RNA silencing. J Biotechnol 151, 151-158.

Reyes, C.A., Zanek, M.C., Velazquez, K., Costa, N., Plata, M.I., Garcia, M.L., 2011b. Generation of Sweet Orange Transgenic Lines and Evaluation of Citrus psorosis virus-derived Resistance against Psorosis A and Psorosis B. J Phytopathol 159, 531-537.

Roberts, M., Minott, D.A., Pinnock, S., Tennant, P.F., Jackson, J.C., 2014. Physicochemical and biochemical characterization of transgenic papaya modified for protection against Papaya ringspot virus. J Sci Food Agr 94, 1034-1038.

Roy, G., Sudarshana, M.R., Ullman, D.E., Ding, S.W., Dandekar, A.M., Falk, B.W., 2006. Chimeric cDNA sequences from Citrus tristeza virus confer RNA silencing-mediated resistance in transgenic Nicotiana benthamiana plants. Phytopathology 96, 819-827.

Roy, S., Banerjee, A., Tarafdar, J., Senapati, B.K., Dasgupta, I., 2012. Transfer of transgenes for resistance to rice tungro into high-yielding rice cultivars through gene-based marker-assisted selection. J Agr Sci 150, 610-618.

Sasaya, T., Nakazono-Nagaoka, E., Saika, H., Aoki, H., Hiraguri, A., Netsu, O., Uehara-Ichiki, T., Onuki, M., Toki, S., Saito, K., Yatou, O., 2014. Transgenic strategies to confer resistance against viruses in rice plants. Front Microbiol 4, 409.

Savenkov, E.I., Valkonen, J.P., 2001. Coat protein gene-mediated resistance to Potato virus A in transgenic plants is suppressed following infection with another potyvirus. J Gen Virol 82, 2275-2278.

Scorza, R., Callahan, A., Levy, L., Damsteegt, V., Webb, K., Ravelonandro, M., 2001. Post-transcriptional gene silencing in plum pox virus resistant transgenic European plum containing the plum pox potyvirus coat protein gene. Transgenic Res 10, 201-209.

Shekhawat, U.K.S., Ganapathi, T.R., Hadapad, A.B., 2012. Transgenic banana plants expressing small interfering RNAs targeted against viral replication initiation gene display high-level resistance to banana bunchy top virus infection. Journal of General Virology 93, 1804-1813.

Shepherd, D.N., Mangwende, T., Martin, D.P., Bezuidenhout, M., Thomson, J.A., Rybicki, E.P., 2007. Inhibition of maize streak virus (MSV) replication by transient and transgenic expression of MSV replication-associated protein mutants. J Gen Virol 88, 325-336.

Sherman, J.M., Moyer, J.W., Daub, M.E., 1998. Tomato spotted wilt virus resistance in chrysanthemum expressing the viral nucleocapsid gene. Plant Dis. 82, 407-414.

Shimizu, T., Nakazono-Nagaoka, E., Uehara-Ichiki, T., Sasaya, T., Omura, T., 2011. Targeting specific genes for RNA interference is crucial to the development of strong resistance to rice stripe virus. Plant Biotechnol J 9, 503-512.

Shin, R., Han, J.H., Lee, G.J., Peak, K.H., 2002. The potential use of a viral coat protein gene as a transgene screening marker and multiple virus resistance of pepper plants coexpressing coat proteins of cucumber mosaic virus and tomato mosaic virus. Transgenic Research 11, 215-219.

Shivaprasad, P.V., Thillaichidambaram, P., Balaji, V., Veluthambi, K., 2006. Expression of full-length and truncated Rep genes from Mungbean yellow mosaic virus-Vigna inhibits viral replication in transgenic tobacco. Virus Genes 33, 365-374.

Singh, A., Taneja, J., Dasgupta, I., Mukherjee, S.K., 2015. Development of plants resistant to tomato geminiviruses using artificial trans-acting small interfering RNA. Mol Plant Pathol 16, 724-734.

Sivamani, E., Brey, C., Dyer, W.E., Talbert, L.E., Qu, R., 2000. Resistance to wheat streak mosaic virus in transgenic wheat expressing the viral replicase (NIb) gene. Molecular Breeding 6, 469-477.

Sivamani, E., Brey, C.W., Talbert, L.E., Young, M.A., Dyer, W.E., Kaniewski, W.K., Qu, R., 2002. Resistance to wheat streak mosaic virus in transgenic wheat engineered with the viral coat protein gene. Transgenic Res 11, 31-41.

Soler, N., Plomer, M., Fagoaga, C., Moreno, P., Navarro, L., Flores, R., Pena, L., 2012. Transformation of Mexican lime with an intron-hairpin construct expressing untranslatable versions of the genes coding for the three silencing suppressors of Citrus tristeza virus confers complete resistance to the virus. Plant Biotechnology Journal 10, 597-608.

Song, Y.Z., Han, Q.J., Jiang, F., Sun, R.Z., Fan, Z.H., Zhu, C.X., Wen, F.J., 2014. Effects of the sequence characteristics of miRNAs on multi-viral resistance mediated by single amiRNAs in transgenic tobacco. Plant Physiol Biochem 77, 90-98.

Sonoda, S., 2003. Analysis of the nucleocapsid protein gene from Tomato spotted wilt virus as target and inducer for posttranscriptional gene silencing. Plant Sci 164, 717-725.

Sonoda, S., Tsumuki, H., 2004. Analysis of RNA-mediated virus resistance by NSs and NSm gene sequences from Tomato spotted wilt virus. Plant Sci 166, 771-778.

Tennant, P., Ahmad, M.H., Gonsalves, D., 2005. Field resistance of coat protein transgenic papaya to Papaya ringspot virus in Jamaica. Plant Disease 89, 841-847.

Tyagi, H., Rajasubramaniam, S., Rajam, M.V., Dasgupta, I., 2008. RNA-interference in rice against Rice tungro bacilliform virus results in its decreased accumulation in inoculated rice plants. Transgenic Research 17, 897-904.

Valarmathi, P., Kumar, G., Robin, S., Manonmani, S., Dasgupta, I., Rabindran, R., 2016. Evaluation of virus resistance and agronomic performance of rice cultivar ASD 16 after transfer of transgene against Rice tungro bacilliform virus by backcross breeding. Virus Genes 52, 521-529.

Vanderschuren, H., Akbergenov, R., Pooggin, M.M., Hohn, T., Gruissem, W., Zhang, P., 2007. Transgenic cassava resistance to African cassava mosaic virus is enhanced by viral DNA-A bidirectional promoter-derived siRNAs. Plant Molecular Biology 64, 549-557.

Vaslin, M.F., Vidal, M.S., Alves, E.D., Farinelli, L., de Oliveira, D.E., 2001. Co-suppression mediated virus resistance in transgenic tobacco plants harboring the 3'-untranslated region of Andean potato mottle virus. Transgenic Res 10, 489-499.

Vassilakos, N., Bem, F., Tzima, A., Barker, H., Reavy, B., Karanastasi, E., Robinson, D.J., 2008. Resistance of transgenic tobacco plants incorporating the putative 57-kDa polymerase read-through gene of Tobacco rattle virus against rub-inoculated and nematode-transmitted virus. Transgenic Res 17, 929-941.

Vasudevan, A., Oh, T.K., Park, J.S., Lakshmi, S.V., Choi, B.K., Kim, S.H., Lee, H.J., Ji, J., Kim, J.H., Ganapathi, A., Kim, S.C., Choi, C.W., 2008. Characterization of resistance mechanism in transgenic Nicotiana benthamiana containing Turnip crinkle virus coat protein. Plant Cell Rep 27, 1731-1740.

Voloudakis, A.E., Aleman-Verdaguer, M.E., Padgett, H.S., Beachy, R.N., 2005. Characterization of resistance in transgenic Nicotiana benthamiana encoding N-terminal deletion and assembly mutants of the tobacco etch potyvirus coat protein. Arch Virol 150, 2567-2582.

Vu, T.V., Choudhury, N.R., Mukherjee, S.K., 2013. Transgenic tomato plants expressing artificial microRNAs for silencing the pre-coat and coat proteins of a begomovirus, Tomato leaf curl New Delhi virus, show tolerance to virus infection. Virus Res 172, 35-45.

Wang, F., Li, W., Zhu, J., Fan, F., Wang, J., Zhong, W., Wang, M.B., Liu, Q., Zhu, Q.H., Zhou, T., Lan, Y., Zhou, Y., Yang, J., 2016. Hairpin RNA Targeting Multiple Viral Genes Confers Strong Resistance to Rice Black-Streaked Dwarf Virus. Int J Mol Sci 17.

Wang, X., Eggenberger, A.L., Nutter, F.W., Hill, J.H., 2001. Pathogen-derived transgenic resistance to soybean mosaic virus in soybean. Molecular Breeding 8, 119-127.

Waterhouse, P.M., Graham, M.W., Wang, M.B., 1998. Virus resistance and gene silencing in plants can be induced by simultaneous expression of sense and antisense RNA. Proc Natl Acad Sci U S A 95, 13959-13964.

Wittner, A., Palkovics, L., Balazs, E., 1998. Nicotiana benthamiana plants transformed with the plum pox virus helicase gene are resistant to virus infection. Virus Res 53, 97-103.

Wu, H.W., Yu, T.A., Raja, J.A.J., Christopher, S.J., Wang, S.L., Yeh, S.D., 2010. Double-Virus Resistance of Transgenic Oriental Melon Conferred by Untranslatable Chimeric Construct Carrying Partial Coat Protein Genes of Two Viruses. Plant Disease 94, 1341-1347.

Xu, L., Song, Y.Z., Zhu, J.H., Guo, X.Q., Zhu, C.X., Wen, F.J., 2009. Conserved Sequences of Replicase Gene-Mediated Resistance to Potyvirus through RNA Silencing. J Plant Biol 52, 550-559.

Yadav, J.S., Ogwok, E., Wagaba, H., Patil, B.L., Bagewadi, B., Alicai, T., Gaitan-Solis, E., Taylor, N.J., Fauquet, C.M., 2011. RNAi-mediated resistance to Cassava brown streak Uganda virus in transgenic cassava. Molecular Plant Pathology 12, 677-687.

Yang, H., Ozias-Akins, P., Culbreath, A.K., Gorbet, D.W., Weeks, J.R., Mandal, B., Pappu, H.R., 2004. Field evaluation of Tomato spotted wilt virus resistance in transgenic peanut (Arachis hypogaea). Plant Dis. 88, 259-264.

Yousaf, S., Rasool, G., Amin, I., Mansoor, S., Saeed, M., 2013. Interference of a Synthetic Rep Protein to Develop Resistance against Cotton Leaf Curl Disease. Int J Agric Biol 15, 1140-1144.

Zanek, M.C., Reyes, C.A., Cervera, M., Pena, E.J., Velazquez, K., Costa, N., Plata, M.I., Grau, O., Pena, L., Garcia, M.L., 2008. Genetic transformation of sweet orange with the coat protein gene of Citrus psorosis virus and evaluation of resistance against the virus. Plant Cell Rep 27, 57-66.

Zhang, X., Li, H., Zhang, J., Zhang, C., Gong, P., Ziaf, K., Xiao, F., Ye, Z., 2011a. Expression of artificial microRNAs in tomato confers efficient and stable virus resistance in a cell-autonomous manner. Transgenic Res 20, 569-581.

Zhang, X., Sato, S., Ye, X., Dorrance, A.E., Morris, T.J., Clemente, T.E., Qu, F., 2011b. Robust RNAi-based resistance to mixed infection of three viruses in soybean plants expressing separate short hairpins from a single transgene. Phytopathology 101, 1264-1269.

Zhang, Z.Y., Fu, F.L., Gou, L., Wang, H.G., Li, W.C., 2010. RNA Interference-Based Transgenic Maize Resistant to Maize Dwarf Mosaic Virus. J Plant Biol 53, 297-305.

Zhang, Z.Y., Wang, Y.G., Shen, X.J., Li, L., Zhou, S.F., Li, W.C., Fu, F.L., 2013. RNA interference-mediated resistance to maize dwarf mosaic virus. Plant Cell Tiss Org 113, 571-578.

Zhang, Z.Y., Yang, L., Zhou, S.F., Wang, H.G., Li, W.C., Fu, F.L., 2011c. Improvement of resistance to maize dwarf mosaic virus mediated by transgenic RNA interference. J Biotechnol 153, 181-187.
