# Supplementary material for: Small RNA Based Genetic Engineering for Plant Viral Resistance: Application in Crop Protection
Source: Front Microbiol. 2017 Jan 23;8:43. doi: 10.3389/fmicb.2017.00043 (PMC5253543; doi:10.3389/fmicb.2017.00043)
Supplement: Supplementary file 2 [file Table_2.DOCX]

Table S2 List of viral resistant crops approved for commercial release and are generated with SRGE technology

| **Common name** | **Latin name** | **Event name** | **Virus** | **Gene introduced** | **Function** | **Developer** | **Country** |
| --- | --- | --- | --- | --- | --- | --- | --- |
| Bean | [*Phaseolus vulgaris*](http://www.isaaa.org/gmapprovaldatabase/crop/default.asp?CropID=3) | EMBRAPA 5.1 | BGMV | ac1 (sense and antisense) | Silencing against BGMV | EMBRAPA | Brazil |
| Papaya | [*Carica papaya*](http://www.isaaa.org/gmapprovaldatabase/crop/default.asp?CropID=11) | Jan-55 | PRSV | [prsv_cp](http://www.isaaa.org/gmapprovaldatabase/gene/default.asp?GeneID=73&Gene=prsv_cp) | "PDR"* to PRSV | Cornell University and University of Hawaii | Canada, Japan, USA |
| Papaya | [*Carica papaya*](http://www.isaaa.org/gmapprovaldatabase/crop/default.asp?CropID=11) | 63-1 | PRSV | [prsv_cp](http://www.isaaa.org/gmapprovaldatabase/gene/default.asp?GeneID=73&Gene=prsv_cp) | "PDR" to PRSV |  | USA |
| Papaya | [*Carica papaya*](http://www.isaaa.org/gmapprovaldatabase/crop/default.asp?CropID=11) | Huanong No. 1 | PRSV | [prsv_rep](http://www.isaaa.org/gmapprovaldatabase/gene/default.asp?GeneID=92&Gene=prsv_rep) | Silencing against PRSV | South China Agricultural University | China |
| Papaya | [*Carica papaya*](http://www.isaaa.org/gmapprovaldatabase/crop/default.asp?CropID=11) | X17-2 | PRSV | [prsv_cp](http://www.isaaa.org/gmapprovaldatabase/gene/default.asp?GeneID=73&Gene=prsv_cp) | "PDR" to PRSV | University of Florida | USA |
| Plum | *Prunus domestica* | C-5 | PPV | [ppv_cp](http://www.isaaa.org/gmapprovaldatabase/gene/default.asp?GeneID=74&Gene=ppv_cp) | "PDR" to PPV | USDA-ARS | USA |
| Potato | *Solanum tuberosum L.* | HLMT15-15 | PVY | [pvy_cp](http://www.isaaa.org/gmapprovaldatabase/gene/default.asp?GeneID=67&Gene=pvy_cp) | "PDR" to PVY | Monsanto Company | USA |
| Potato | *Solanum tuberosum L.* | HLMT15-3 | PVY | [pvy_cp](http://www.isaaa.org/gmapprovaldatabase/gene/default.asp?GeneID=67&Gene=pvy_cp) | "PDR" to PVY | Monsanto Company | USA |
| Potato | *Solanum tuberosum L.* | HLMT15-46 | PVY | [pvy_cp](http://www.isaaa.org/gmapprovaldatabase/gene/default.asp?GeneID=67&Gene=pvy_cp) | "PDR" to PVY | Monsanto Company | USA |
| Potato | *Solanum tuberosum L.* | RBMT15-101 | PVY | [pvy_cp](http://www.isaaa.org/gmapprovaldatabase/gene/default.asp?GeneID=67&Gene=pvy_cp) | "PDR" to PVY | Monsanto Company | Australia, Canada, Japan, Mexico, New Zealand, Philippines, South Korea, USA |
| Potato | *Solanum tuberosum L.* | RBMT21-129 | PLRV | plrv_orf1orf2 | Silencing against PLRV | Monsanto Company |  |
| Potato | *Solanum tuberosum L.* | RBMT21-152 | PLRV | plrv_orf1orf3 | Silencing against PLRV | Monsanto Company | USA |
| Potato | *Solanum tuberosum L.* | RBMT21-350 | PLRV | plrv_orf1orf4 | Silencing against PLRV | Monsanto | Canada, Japan, Mexico, New Zealand, Philippines, South Korea, USA |
| Potato | *Solanum tuberosum L.* | RBMT22-082 | PLRV | plrv_orf1orf5 | Silencing against PLRV | Monsanto Company |  |
| Potato | *Solanum tuberosum L.* | RBMT22-186 | PLRV | plrv_orf1orf6 | Silencing against PLRV | Monsanto Company | USA |
| Potato | *Solanum tuberosum L.* | RBMT22-238 | PLRV | plrv_orf1orf7 | Silencing against PLRV | Monsanto Company | USA |
| Potato | *Solanum tuberosum L.* | RBMT22-262 | PLRV | plrv_orf1orf8 | Silencing against PLRV | Monsanto Company | USA |
| Potato | *Solanum tuberosum L.* | SEMT15-07 | PVY | [pvy_cp](http://www.isaaa.org/gmapprovaldatabase/gene/default.asp?GeneID=67&Gene=pvy_cp) | "PDR" to PVY | Monsanto Company | USA |
| Potato | *Solanum tuberosum L.* | SEMT15-02 | PVY | [pvy_cp](http://www.isaaa.org/gmapprovaldatabase/gene/default.asp?GeneID=67&Gene=pvy_cp) | "PDR" to PVY | Monsanto Company | Canada, Japan, Mexico, New Zealand, Philippines, South Korea, USA |
| Potato | *Solanum tuberosum L.* | SEMT15-15 | PVY | [pvy_cp](http://www.isaaa.org/gmapprovaldatabase/gene/default.asp?GeneID=67&Gene=pvy_cp) | "PDR" to PVY | Monsanto Company |  |
| Squash | *Cucurbita pepo* | CZW3 | CMV | cmv/zymv/wmv cp | "PDR" to CMV | Seminis Vegetable Seeds (Canada) and Monsanto Company (Asgrow) | Canada, USA |
| Squash | *Cucurbita pepo* | ZW20 | ZYMV | zymv_cp/wmv cp | "PDR" to ZYMV |  | USA |
| Sweet pepper | *Capsicum annuum* | PK-SP01 | CMV | cmv_cp | "PDR" to CMV | Beijing University | China |
| Tomato | *Lycopersicon esculentum* | PK-TM8805R | CMV) | cmv_cp | "PDR" to CMV | Beijing University | China |
| Sources: | <http://www.isaaa.org/> |  |  |  |  |  |  |
